# Targeted deletion of von-Hippel-Lindau in the proximal tubule conditions the kidney against early diabetic kidney disease

**DOI:** 10.1038/s41419-023-06074-7

**Published:** 2023-08-26

**Authors:** Madlen Kunke, Hannah Knöfler, Eileen Dahlke, Luis Zanon Rodriguez, Martina Böttner, Alexey Larionov, Makhabbat Saudenova, Gerrit M. Ohrenschall, Magdalena Westermann, Stefan Porubsky, Joana P. Bernardes, Robert Häsler, Jean-Luc Magnin, Hermann Koepsell, François Jouret, Franziska Theilig

**Affiliations:** 1 Institute of Anatomy, Christian Albrechts-University Kiel, Kiel, Germany; 2 Institute of Anatomy, Department of Medicine, University of Fribourg, Fribourg, Switzerland; 3 Institute of Pathology, University of Mainz, Mainz, Germany; 4 Department of Dermatology and Allergy, University Hospital Schleswig-Holstein, Kiel, Germany; 5 Laboratoire HFR- Hôpital Cantonal, Fribourg, Switzerland; 6 Institute of Anatomy and Cell Biology, Julius-Maximilians-University of Würzburg, Würzburg, Germany; 7 Groupe Interdisciplinaire de Génoprotéomique Appliquée (GIGA), Cardiovascular Sciences, University of Liège (ULiège), Liège, Belgium; 8 Division of Nephrology, CHU of Liège, University of Liège (CHU ULiège), Liège, Belgium

**Keywords:** Kidney, Nephrons

## Abstract

Diabetic kidney disease (DKD) is the leading cause of end-stage renal disease. Glomerular hyperfiltration and albuminuria subject the proximal tubule (PT) to a subsequent elevation of workload, growth, and hypoxia. Hypoxia plays an ambiguous role in the development and progression of DKD and shall be clarified in our study. PT-von-Hippel-Lindau (*Vhl*)-deleted mouse model in combination with streptozotocin (STZ)-induced type I diabetes mellitus (DM) was phenotyped. In contrary to PT-*Vhl*-deleted STZ-induced type 1 DM mice, proteinuria and glomerular hyperfiltration occurred in diabetic control mice the latter due to higher nitric oxide synthase 1 and sodium and glucose transporter expression. PT *Vhl* deletion and DKD share common alterations in gene expression profiles, including glomerular and tubular morphology, and tubular transport and metabolism. Compared to diabetic control mice, the most significantly altered in PT *Vhl*-deleted STZ-induced type 1 DM mice were *Ldc-1*, regulating cellular oxygen consumption rate, and *Zbtb16*, inhibiting autophagy. Alignment of altered genes in heat maps uncovered that *Vhl* deletion prior to STZ-induced DM preconditioned the kidney against DKD. HIF-1α stabilization leading to histone modification and chromatin remodeling resets most genes altered upon DKD towards the control level. These data demonstrate that PT HIF-1α stabilization is a hallmark of early DKD and that targeting hypoxia prior to the onset of type 1 DM normalizes renal cell homeostasis and prevents DKD development.

## Introduction

The incidence of diabetes mellitus (DM) and its complications, including diabetic kidney disease (DKD), increases worldwide. DKD has been traditionally viewed as a microvascular disorder clustered with retinopathy and neuropathy. Currently, it is widely accepted that tissue-specific manifestation results from the same pathogenic glucose-driven process that occurs at susceptible sites in the body [1]. One-third of patients with DM develop DKD whereas almost all patients develop some degree of retinopathy, suggesting additional risk factors beyond hyperglycemia. Among them, hemodynamic factors, activation of the renin-angiotensin-aldosterone system (RAAS), oxidative stress, and hypoxia have been implicated in DKD [1]. DKD progresses in defined stages and is the major cause of end-stage renal disease (ESRD). The pathogenesis of DKD has been mostly characterized by glomerular pathological changes [2]. A “tubule-centric” view has progressively arisen [3] since the diabetic proximal tubule (PT) shows morphological and metabolic alterations before the onset of glomerular alterations and might therefore be the primary pathological event in DKD [3, 4].

The PT is adjacent to the glomerulus and is immediately exposed to the ultrafiltrate containing extensive amounts of key body components. Adapted to the body’s needs, the PT physiologically reabsorbs nutrients, small proteins, electrolytes, and trace elements. Equipped with a great membrane surface for transepithelial transport and abundant mitochondria, the PT is the most metabolically active part of the kidney. In DKD, the PT is exposed to nutrient overload due to glomerular hyperfiltration. Inhibitors of PT Na^+^-glucose uptake (gliflozins) have recently revolutionized the treatment of DKD by slowing down the decline in kidney function and the progression to ESRD [5–8]. The precise molecular mechanism underlying renal-protective effects of sodium-glucose co-transporter 2 (SGLT2) inhibition is not completely understood but most probably involves hemodynamic effects [9], and the attenuation of hypoxia and inflammation [6, 10].

Renal hypoxia is frequently observed in DKD [11]. The most important known cellular oxygen-sensing machinery is hypoxia-inducible factors (HIF) α and β, creating a functional complex for the initiation of the transcription of hypoxia-related genes [12]. HIF-1α is stabilized upon hypoxic conditions but rapidly degraded upon normoxia via proline hydroxylation by prolyl hydroxylase domain enzymes and polyubiqutination by an E3 ubiquitin ligase composed of von-Hippel-Lindau protein (pVHL)-Elongin BC-CUL2 complex. The requirement of several enzymes and cofactors for HIF degradation makes it possible to stabilize HIF in normoxic conditions with inhibition or decrease of any of the degradation components.

To test the hypothesis that PT hypoxia contributes to DKD, we used mice with *Vhl* deletion upon Cre recombinase expression under the control of the SGLT2 promoter to study the effects of pVHL-mediated HIF-1α stabilization mimicking PT hypoxia in DKD. Employing transcriptomics, biochemical assessments of membrane transporters and signaling proteins, and morphological approaches, we conducted a comprehensive study to better understand the role of PT hypoxia in the early stages of DKD.

## Results

### Genetic proximal tubular *Vhl* deletion prevents diabetic glomerular hyperfiltration and proteinuria

**Fig. 1:**
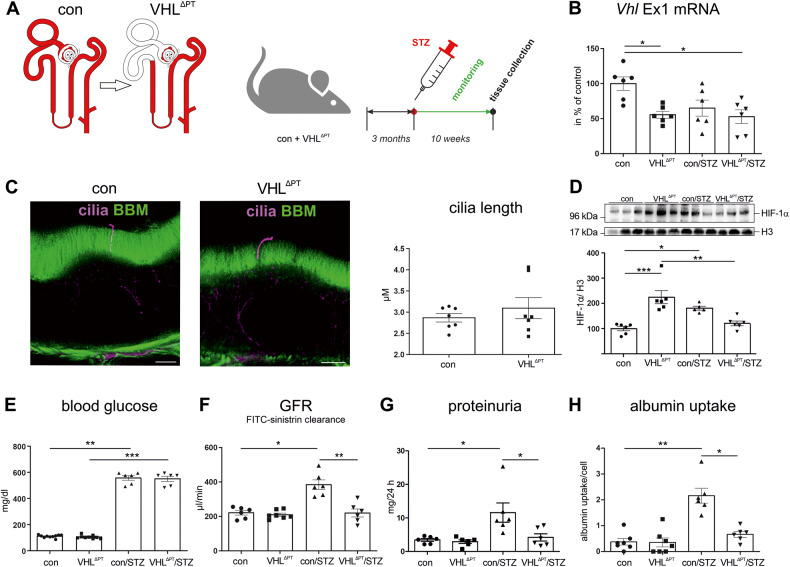
Deletion of *Vhl* expression in early proximal tubule segments (S1/2) and renal function after induction of type 1 diabetes mellitus. **A** Scheme illustrating deletion of the von-Hippel-Lindau (*Vhl*) gene under the sodium-glucose co-transporter 2 (*Sglt2*) promoter in S1 and S2 segments of the proximal tubule and time-line of the experiment. **B** Semi-quantitative analysis of *Vhl* mRNA expression derived from Exon 1 in S1/2 segments of control, VHL^ΔPT^, con/STZ, and VHL^ΔPT^/STZ. Arithmetic means ± SEM of *n* = 6 per group; *P < 0.05. **C** STED images of cilia from proximal tubules obtained from control and VHL^ΔPT^ and morphometric analysis of cilia length. BBM, brush border membrane. Arithmetic means ± SEM of *n* = 7 per group. Scale bar = 2 µm. Nonparametric Mann–Whitney-*U*-test. **D** Western blot of renal nuclear HIF-1α abundance and densitometric evaluation of control, VHL^ΔPT^, con/STZ, and VHL^ΔPT^/STZ with histone H3 as reference. Arithmetic means ± SEM of *n* = 5–6 per group; *P < 0.05, **P < 0.01, ***P < 0.001. **E**–**H** Renal function data: blood glucose (**E**), glomerular filtration rate (GFR) determined by FITC-sinistrin plasma kinetic measurements (**F**), proteinuria of 24 h urine collection (**G**), and proximal tubular albumin uptake (**H**) of control, VHL^ΔPT^, con/STZ, and VHL^ΔPT^/STZ. Arithmetic means ± SEM of *n* = 6–7 per group; *P < 0.05, **P < 0.01, ***P < 0.001. **B**–**G** Each point represents an individual mouse. **B**, **D**, **E**–**G** Nonparametric Kruskal–Wallis with Dunn’s post-test.

To examine the effects of PT hypoxia reflected as VHL-mediated HIF-1α stabilization in conditions of type 1 diabetes mellitus, we generated mice with deletion of *Vhl* in the early PT S1/2 segments by cross-breeding *Vhl*^flox^ mice, termed control (con), with *Sglt2*^cre^ mice, termed VHL^∆PT^. Diabetes was induced in control and VHL^ΔPT^ mice by administration of STZ, termed con/STZ and VHL^ΔPT^/STZ. Mice were monitored for 10 weeks post STZ treatment (Fig. 1A). Determining the degree of *Vhl* knockout by quantifying the *Vhl*-Exon 1-derived mRNA expression in SGLT2-positive PT cells revealed a 44.5 ± 5.2% and a 47.3 ± 9.6% deletion of *Vhl* in S1/2 segments in VHL^ΔPT^ and VHL^∆PT^/STZ, respectively (Fig. 1B). Cilia length of PT cells remained normal upon PT *Vhl* deletion (Fig. 1C, Supplemental Fig. 1A). HIF-1α, targeted by VHL ubiquitination for proteasomal degradation, was found to be strongly increased in VHL^ΔPT^ and in con/STZ compared to controls (Fig. 1D). HIF-1α was significantly reduced in VHL^ΔPT^/STZ compared to VHL^ΔPT^. HIF-1α downstream targets such as VEGF and GLUT1 were observed in the proximal tubule in a mosaic-dependent fashion in VHL^ΔPT^ and VHL^ΔPT^/STZ and total protein abundance changed according to nuclear HIF-1α expression level (Supplemental Fig. 1B, C). Renal function was assessed. Plasma analysis of mice 10 weeks after STZ treatment revealed significantly higher blood glucose levels in both diabetic groups to a similar extent (Fig. 1E). The GFR in conscious mice 10 weeks after STZ treatment, revealed an augmented GFR in con/STZ, which is consistent with glomerular hyperfiltration, an early clinical hallmark in DM (Fig. 1F). By contrast, VHL^ΔPT^/STZ mice did not show glomerular hyperfiltration. Analysis of samples revealed significantly higher protein excretion in 24-h urine collections at 10 weeks after STZ treatment and increased proximal tubular albumin uptake in con/STZ (Fig. 1G, H). In addition, greater urinary flow rate, urinary glucose, and phosphate excretion (Supplemental Fig. 1D) were increased upon STZ treatment compared to the respective controls. Urinary sodium excretion was increased only in VHL^ΔPT^/STZ compared to VHL^ΔPT^. To evaluate kidney morphology, PAS-stained sections were analyzed for glomerular and tubulointerstitial injury (Supplemental Fig. 1E–G). In VHL^ΔPT^ and VHL^ΔPT^/STZ, only little glomerular alterations and no tubulointerstitial injury were identified. In con/STZ, high glomerular and tubular injury was observed.

### Differentially altered expression of tubular sodium and glucose transporters and NOS1 upon genetic proximal tubular *Vhl* deletion and after STZ-induced type 1 diabetes

**Fig. 2:**
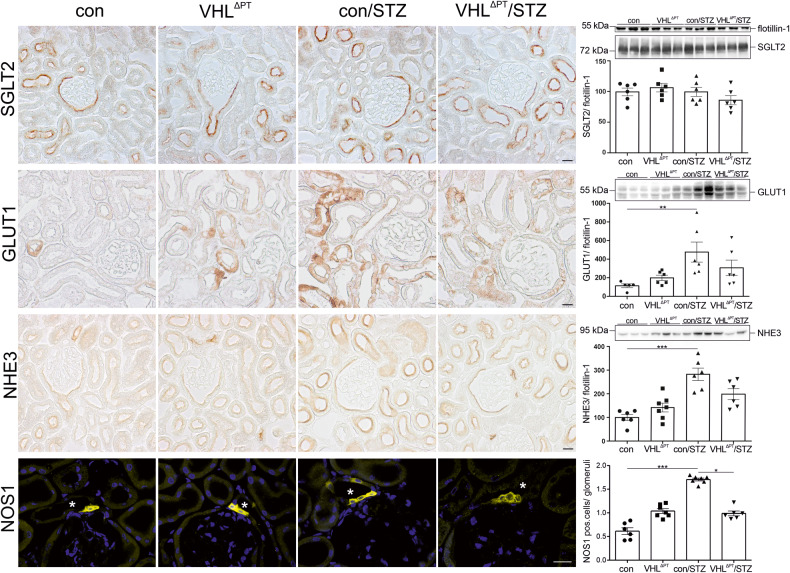
Sodium and glucose transporter of the proximal and distal tubule and nitric oxide synthase 1. (Left) Representative images of immunohistochemical staining of sodium-glucose co-transporter 2 (SGLT2), glucose transporter-1 (GLUT1), sodium hydrogen exchanger-3 (NHE3), and nitric oxide synthase 1 (NOS1) obtained from control, VHL^ΔPT^, con/STZ and VHL^ΔPT^/STZ. Asterisks indicate macula densa cells located in the pars recta of the distal tubule. Scale bar = 20 µm. (Right) Representative blots and densitometrical evaluation of western blots of SGLT2, GLUT1, NHE3, and semi-quantitative evaluation of NOS1 obtained from control, VHL^ΔPT^, con/STZ, and VHL^ΔPT^/STZ. Flotillin-1 was used as a blot reference. Arithmetic means ± SEM of *n* = 5–7 per group; *P < 0.05, **P < 0.01, ***P < 0.001. (Right) Each band/point represents an individual mouse. Nonparametric Kruskal–Wallis with Dunn’s post test.

To study the proteins classically involved in the DM-associated glomerular hyperfiltration and higher solute excretion, the expression of PT and thick ascending limb (TAL) sodium and glucose transporters and nitric oxide synthase 1 (NOS1) were determined by immunohistochemistry and western blot analyses (Fig. 2). Sodium-glucose co-transporter 2 (SGLT2), expressed in S1 and S2 segments, remained unaltered. The glucose transporter-1 (GLUT1) expressed most prominently in the basolateral membrane of distal tubules and connecting tubules/ collecting ducts in controls demonstrated additional expression in PT in VHL^ΔPT^ and to a lesser extent VHL^ΔPT^/STZ (Supplemental Fig. 1C) which is in agreement with being a downstream target of HIF-1α, whereas total protein GLUT1 protein was higher in con/STZ. NHE3, localized apically to the PT and TAL, were higher in con/STZ. Levels of NKCC2 expressed apically in TAL were significantly higher in con/STZ (Supplemental Fig. 2A, B). Thus, the higher expression of sodium transporters in con/STZ support the tubule-centered model where low sodium concentrations in the filtrate eventually reach the macula densa, thereby leading to a decline in the tubuloglomerular feedback (TGF) mechanism and subsequent glomerular hyperfiltration. This pathological circuit was not observed in VHL^ΔPT^/STZ. Increased NOS1 expression and activity in diabetic mice and humans have been demonstrated to control the GFR in DKD. We, therefore, tested NOS1 expression in our experimental groups. NOS1, specifically expressed in the macula densa cells of the distal tubule, was significantly higher in con/STZ compared to control and VHL^ΔPT^/STZ and may also account for the glomerular hyperfiltration found only in con/STZ (Fig. 2).

### *Vhl* deletion induces similar alterations as in STZ-induced diabetes and preconditions against STZ-induced DKD

**Fig. 3:**
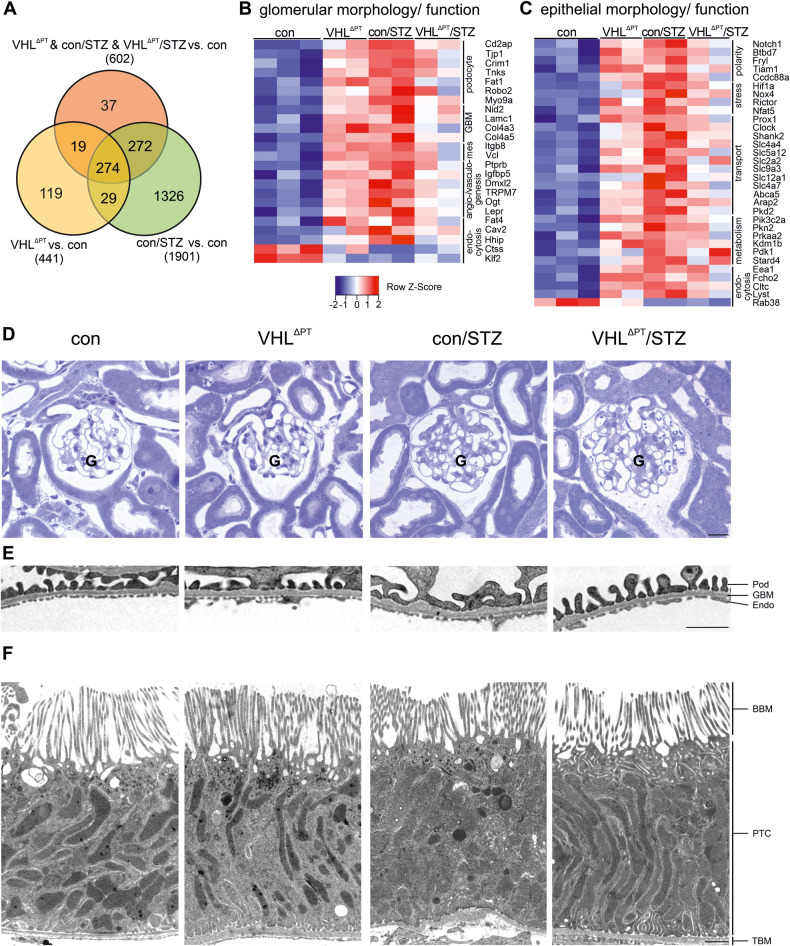
*Vhl* deletion preconditions against STZ-induced DKD development. **A** Venn diagram of the number of genes altered comparing VHL^ΔPT^ vs. control, con/STZ vs. control, and VHL^ΔPT^ + con/STZ + VHL^∆PT^/STZ vs. control based on filter criteria DESeq *P*-values < 0.05, fold-change >1.5. **B** and **C** Heat maps of genes significantly altered in VHL^ΔPT^ & con/STZ & VHL^ΔPT^/STZ vs. control, filter criteria used DESeq *P*-values < 0.05, fold-change > 1.5. **B** Altered genes sorted depending on known glomerular function related to podocyte, basement membrane (GBM), mesangial cells (mes), angio/vasculogenesis, and endocytosis. **C** Altered genes are sorted depending on known tubular function related to cell polarity, cell stress, transport, metabolism, and endocytosis. **D** Light microscopy of semi-thin sections showing representative glomeruli from each group. Glomeruli are marked by a “G”. The number of capillary profiles is higher upon *Vhl* deletion and diabetes induction and augmented glomerular size is visible upon diabetes induction. Scale bar = 20 µm. **E** and **F** Electron microscopy of ultra-thin sections showing representative images of glomerular filtration barrier (**E**) and of proximal tubule cells from S1 segments (**F**). Note, thickening of glomerular basement membrane (GBM) upon *Vhl* deletion and diabetes induction, foot process effacement of podocytes (Pod) only in the con/STZ group, and augmented epithelial height and tubular basement membrane (TBM) height upon *Vhl* deletion and STZ-induced diabetes. Scale bar = 500 nm and 2 µm, respectively. Glomerular endothelial cells (Endo), brush border membrane of proximal tubule cells (BBM), and proximal tubular cell (PTC).

RNA sequencing of kidney samples from all experimental groups was performed (Supplemental Table 1). To see the impact of *Vhl* deletion—and subsequent HIF-1α stabilization—on the development of DKD, we analyzed the gene expression profiles of the commonly regulated genes of VHL^ΔPT^ & con/STZ & VHL^ΔPT^/STZ mice: ~30% of altered RNA from con/STZ are related to HIF-1α stabilization (overlap of circles, Fig. 3A). Of the commonly altered genes from VHL^ΔPT^ & con/STZ & VHL^ΔPT^/STZ mice, we selected those which were published being relevant for glomerular alterations including podocyte function, glomerular basement membrane thickening, mesangial protein expression, angio- and vasculogenesis and endocytosis (Fig. 3B). Again, VHL^ΔPT^/STZ mice showed the least changes. In detail, VHL^ΔPT^ and STZ-induced diabetes demonstrated (i) higher *Tjp1* (ZO-1) levels, which is consistent with results obtained from human renal biopsies from patients with early DKD [13]; (ii) higher *Nid2*, *Lamc1*, *Col4a3*, *Col4a5*, which is consistent with thickening of the GBM; (iii) higher *Itgb8* and *Vcl*, which is consistent with mesangial proliferation; (iv) higher *Ptprb* and *Ogt*, which is consistent with increased angiogenesis and; (v) lower *Ctss* levels, as observed in renal biopsies from patients with diabetes [14]. A similar picture was obtained by grouping genes for epithelial function, including cell polarity, stress, transport, metabolism, and endocytosis with the strongest differences in con/STZ. VHL^ΔPT^ and VHL^ΔPT^/STZ mice were less altered (Fig. 3C). In details, we observed higher mRNA levels for *Notch1*, *Btbd7*, *Fryl*, which are important genes for renal development; higher *Hif-1a*, *Nox4*, and *Rictor*, which are hypoxia- and stress-induced genes; increased levels of genes regulating epithelial transport, such as kinases and transporters; and higher levels of genes important for endocytosis such as *Cltc* for clathrin and *Eea1* for early endosomes. These results show that *Vhl* deletion induces changes in gene expression, which are regulated in a similar direction as found in DKD. Induction of STZ-mediated type 1 diabetes in VHL^ΔPT^/STZ mice was associated with a milder phenotype of DKD, suggesting that the *Vhl* deletion preconditions the kidney against DKD. Related proteins to the genes of Fig. 3B, C, may affect epithelial and glomerular morphology. Therefore, we performed a detailed renal morphology evaluation. Morphometric analyses of glomeruli from semi-thin sections demonstrate that STZ treatment in con/STZ and VHL^ΔPT^/STZ mice caused an increase in glomerular size presented as areas of Bowman capsule and tuft (Fig. 3D and Table 1). Increased glomerular angiogenesis as observed by greater amounts of capillaries per tuft and thickening of glomerular basement membrane (GBM) were found in VHL^ΔPT^, con/STZ, and VHL^ΔPT^/STZ (Fig. 3D, E and Table 1). Podocyte foot process effacement was observed in con/STZ but not in VHL^ΔPT^/STZ mice (Fig. 3E and Table 1). To study if changes in the PT in addition to glomerular structures occurred, PT profiles of S1 and S2 segments were morphometrically analyzed. An increase in epithelial height and thickening of the tubular basement membrane, both morphological signs of early DKD, augmented in VHL^ΔPT^, con/STZ, and VHL^ΔPT^/STZ mice (Fig. 3F and Table 1).

**Table 1:** Morphometric analysis of glomeruli and proximal tubule cells.

	Control	VHL^ΔPT^	con/STZ	VHL^ΔPT^/ STZ
Glomerular morphometry				
Bowman capsule area in µm^2^	5387 ± 70	5588 ± 189	6378 ± 307*	6793 ± 324*
Tuft area in µm^2^	4276 ± 20	4443 ± 134	5332 ± 332**	5635 ± 346*
capillary area/ tuft area	0.41 ± 0.01	0.45 ± 0.01*	0.51 ± 0.04*	0.52 ± 0.04*
GBM thickness in nm	109.2 ± 9.9	133.1 ± 10.8*	136.0 ± 12.6*	143.2 ± 13.1*
Podocyte foot processes per GMB length in n/µm	3.24 ± 0.24	3.19 ± 0.12	2.94 ± 0.15*	3.18 ± 0.19
Tubular morphometry				
Epithelial height in µm	4.7 ± 0.18	5.78 ± 0.84*	5.88 ± 0.85*	5.65 ± 0.58*
Basement membrane height in nm	80.4 ± 13.5	125.8 ± 16.3*	115.2 ± 35.4	124.8 ± 9.4*
Microvilli length in µm	1.6 ± 0.26	1.5 ± 0.18	1.4 ± 0.14	1.4 ± 0.13
Invagination/surface in n/µm	23.6 ± 4.1	24.2 ± 3.7	28.6 ± 2.2	29.4 ± 6.5

Quantitative analysis of light and electron microscopy of areas from Bowman´s capsule and tuft, capillary per tuft, and glomerular basement membrane (GBM) thickness, podocyte foot processes per GBM length, proximal tubule S1/2 epithelial height, tubular basement membrane thickness, microvilli length and the number of invaginations per cell surface. Arithmetic means ± SEM of *n* = 6 per group.

*P < 0.05, **P < 0.01.

### Dysbalanced VEGF expression and glomerular VEGF backflow

**Fig. 4:**
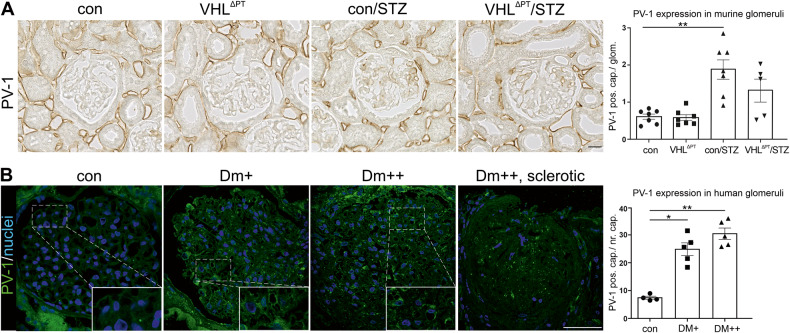
Reduced glomerular VEGF backflow. **A** and **B** Representative images and semi-quantitative analysis of PV-1 positive capillaries per glomeruli of control, VHL^ΔPT^, con/STZ, and VHL^ΔPT^/STZ (**A**) and from human biopsies of patients with interstitial nephritis (control), mild DM (DM + ), moderate DM (DM++) and severe DM (DM + ++). **B**. As control biopsies taken from patients with interstitial nephritis were used. Arithmetic means ± SEM of *n* = 4–7 per group; *P < 0.05, **P < 0.01. Scale bar = 20 µm (**A**) and scale bar = 50 µm (**B**). **A** and **B** Each point represents an individual mouse or patient. Nonparametric Kruskal–Wallis with Dunn’s post-test.

Altered VEGF levels were suggested to be associated with glomerular growth and morphologic changes typical of glomerular DKD [15, 16]. Therefore, we determined glomerular VEGF expression level and VEGF backflow in our mouse model and in biopsies of human kidneys with various degrees of DKD. In mice, no significant difference in mean glomerular VEGF fluorescence intensity was observed (Supplemental Fig. 3A). Mean glomerular VEGF fluorescence intensity of biopsies from DKD kidneys with various degrees demonstrated reduced VEGF expression in severe DM (Supplemental Fig. 3B). Reduced VEGF backflow presented as increased number of plasmalemma vesicles-associated protein-1 (PV-1) positive capillaries with diaphragmed endothelial fenestrae was found in con/STZ and in mild and moderate DM (Fig. 4A, B). To gain mechanistic insight into the reduced VEGF expression level in severe DM, we cultured podocytes for 6 and 24 h in hypoxia and high-glucose conditions. Whereas no alteration was observed after 6 h, a significant decline in *Vegf* mRNA expression was encountered after 24 h (Supplemental Fig. 3C). In comparison, mpkCCD_c14_ cells (cortical collecting duct cells) showed strongly increased *Vegf* mRNA abundance (Supplemental Fig. 3D).

### *Vhl* deletion augments or reduces gene expression known to result in an amelioration of DKD

We aimed to identify the genes differentially expressed by *Vhl* deletion, which may participate in the protection against DKD. Among the most increased genes in VHL^ΔPT^ compared to control mice from RNAseq data, we identified *Skil*, *Cxcl14*, *Prox1*, *Irs2*, *Nr3c1*, *Kl*, *Zbtb20*, *Tfpi2,* and *Pfkp* which were presented earlier to improve DKD and reduced expression for *Bmp4*, which was associated with oxidative stress, inflammation and endothelial dysfunction (Supplemental Fig. 4A). The expression of the glucocorticoid receptor (*Nr3c1*) and klotho were verified at the protein level, thereby confirming RNA sequencing results (Supplemental Fig. 4B, C).

### *Vhl* deletion prior to type 1 DM prevents total gene alteration in DKD by histone modification

**Fig. 5:**
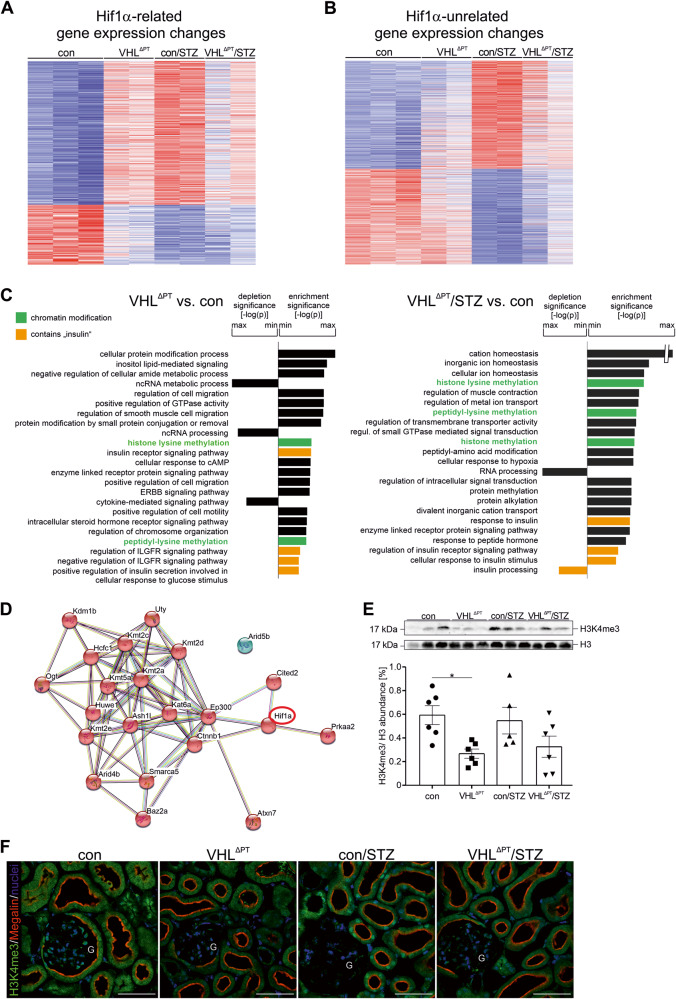
Proximal tubular *Vhl* deletion resets all genes altered upon induction of DKD by reduced H3K4me3 histone modification. **A** and **B** Heat maps of significantly commonly altered genes of VHL^ΔPT^ + con/STZ + VHL^ΔPT^/STZ (HIF-1α related) vs. control (**A**) and remaining HIF-1α unrelated genes altered in con/STZ vs. control (**B**). Filter criteria DESeq *P*-values < 0.05, fold-change > 1.5. **C** Gene ontology analysis of VHL^ΔPT^ and VHL^ΔPT^/STZ vs. control; top 23 biological processes enriched/depleted in the corresponding comparisons. In addition, processes containing the term “chromatin modification” are presented in green, and “insulin“is shown in orange, if significant. **D** STRING network analysis of Gene ontology analysis underlying genes, including *Ash1l, Ogt, Kmt2a, Uty, Kmt2d, Kmt2c, Ep300, Kat6a, Arid4b, Prkaa2, Kmt2e, Kdm5a, Kdm1b, Arid5b, Baz2a, Huwe1, Atxn7*. *Hif-1α* is identified as a functional interaction partner (red circle). **E** and **F** Representative blot, densitometrical evaluation, and immunohistochemical images of H3K4me3 double stained with megalin to mark proximal tubules obtained from control, VHL^ΔPT^, con/STZ and VHL^ΔPT^/STZ with histone H3 as reference. Arithmetic means ± SEM of *n* = 5–7 per group; *P < 0.05. Scale bar = 50 µm. **F** G: glomeruli. Each point represents an individual mouse. Nonparametric Kruskal–Wallis with Dunn’s post-test.

As we observed a rather unchanged gene expression in VHL^ΔPT^/STZ compared to con/STZ of the commonly altered RNA (Fig. 3B, C), we performed a detailed analysis of RNA sequencing results. In con/STZ, 1901 genes were altered. Among them, we identified 602 genes that were commonly regulated upon *Vhl* deletion and were therefore considered to be HIF-1α related. Heat map analysis of the HIF-1α related genes revealed a normalized gene expression profile in VHL^ΔPT^/STZ compared to con/STZ and also to VHL^ΔPT^ (Fig. 5A) strongly supporting *Vhl* deletion-induced preconditioning. We also analyzed the remaining HIF-1α unrelated 1326 gene expressions altered in con/STZ, and the generation of a heat map of all 1326 genes and all groups revealed surprisingly again a rather normalized gene expression profile of VHL^ΔPT^/STZ compared to con/STZ (Fig. 5B). These data demonstrate that *Vhl* deletion prior type 1 DM induction prevents per se DM-associated changes in gene expression responsible for DKD development. We further performed Gene ontology enrichment analysis and found especially in the VHL^ΔPT^ and VHL^ΔPT^/STZ groups, histone lysine modification, peptidyl-lysine methylation, and histone modification among the strongest Gene ontology terms altered (Fig. 5C and Supplemental Fig. 5). Furthermore, STRING network analysis of the Gene ontology term underlying genes validated the interaction between the genes enriched in the Gene ontology term histone modification and in addition revealed HIF-1α as functional interaction partner, showing that preconditioning is related to HIF-1α stabilization (Fig. 5D). To analyze histone modification in more detail, we performed western blot and immunohistochemical analyses of trimethylated H3, a modification which was associated with DKD progression (H3K4me3, Fig. 5E, F). H3K4me3 expression level and its nuclear staining were strongly reduced in VHL^ΔPT^ compared to the control. Note the reduction in glomeruli albeit the PT-specific *Vhl* deletion.

### Significantly altered genes between con/STZ and VHL^∆PT^/STZ mice

**Fig. 6:**
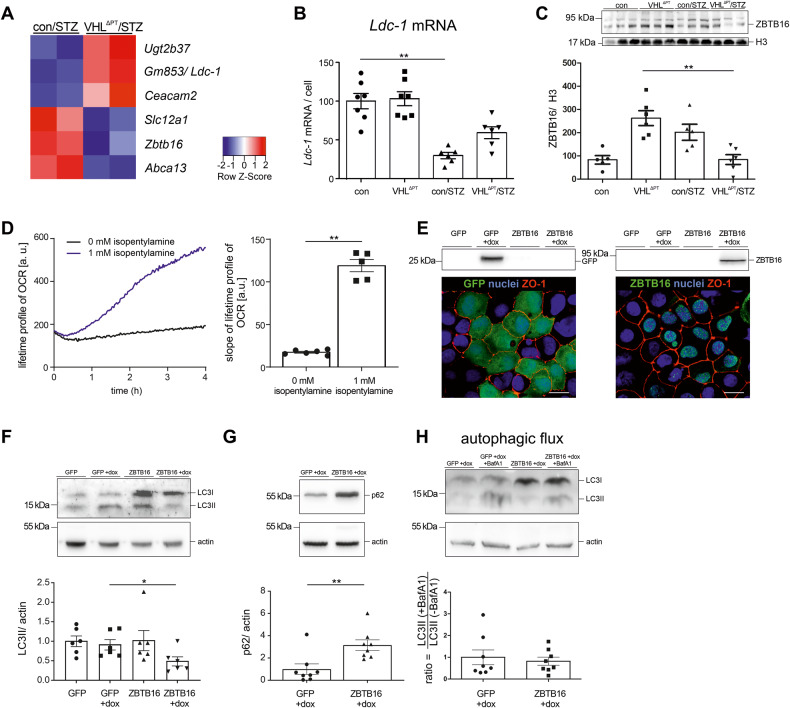
Significantly altered genes between con/STZ and VHL^ΔPT^/STZ. **A** Heat map of genes significantly changed between con/STZ and VHL^ΔPT^/ STZ based on filter criteria DESeq *P*-values < 0.05, fold-change > 1.5. **B** and **C** Analysis of *Ldc-1* mRNA per cell (**B**) and western blot of nuclear fractions and densitometrical analysis of ZBTB16 with histone H3 as reference (**C**). Arithmetic means ± SEM of *n* = 5–7 per group; **P < 0.01. Each point or band represents an individual mouse. Nonparametric Kruskal–Wallis with Dunn’s post test. **D** Cellular oxygen consumption rate (OCR) of opossum kidney cells (OKC) treated with 1 mM isopentylamine leading to strongly increased OCR. Arithmetic means ± SEM, *n* = 5–6 independent experiments; **P < 0.01. Nonparametric Mann–Whitney-*U*-test. **E** Green fluorescence protein (GFP) and ZBTB16 western blot and immunohistochemical staining confirming the generation of control (GFP overexpressing) and ZBTB16 overexpressing OKC line. Scale bar = 10 µm. **F** Western blot of LC3 protein and densitometrical evaluation of LC3II. Arithmetic means ± SEM, *n* = 6 independent experiments; *P < 0.05. Nonparametric Kruskal–Wallis with Dunn’s post test. **G** Western blot and densitometrical evaluation of p62 (SQSTM1). Arithmetic means ± SEM, *n* = 8 independent experiments; **P < 0.01. Nonparametric Mann–Whitney-*U*-test. (**H**) Autophagic flux (ratio between LC3II levels in the presence/ absence of bafilomycin A1, BafA1) of control (GFP overexpressing) and ZBTB16 overexpressing OKC. Arithmetic means ± SEM, *n* = 8 independent experiments. Nonparametric Mann–Whitney-*U*-test. **E**–**H** Αctin was used as a reference.

In search of candidate genes most significantly altered between con/STZ and VHL^ΔPT^/STZ mice, and therefore upon possible DKD preconditioning, we identified *Ugt2b37*, *Gm853/ Ldc-1*, *Ceacam2*, *Slc12a1*, *Zbtb16* and *Abca13* (Fig. 6A). Higher expression of *Slc12a1* (coding for NKCC2) confirmed the enhanced NKCC2 protein expression in kidneys of con/STZ (Supplemental Fig. 2A, B), which adds to the underlying mechanism for the augmented GFR in con/STZ (Fig. 1F). We focused on the identification of the role of *Ldc-1*, a leucine-decarboxylase, and the transcription factor ZBTB16 (zinc finger and BTB domain containing 16, also called PLZF) known as DNA sequence-specific transcriptional repressor. Quantification of *Ldc-1* mRNA signals and ZBTB16 protein levels of nuclear fractions confirmed the results of RNA sequencing (Fig. 6B, C). *Ldc-1* was found to be expressed in the PT cells and to a lesser extent in distal parts of the nephron, and ZBTB16 staining demonstrated nuclear expression in glomerular cells and in epithelial cells along the nephron (Supplemental Fig. 6A). LDC-1, belonging to the ornithine decarboxylase family, was shown to specifically decarboxylate L-leucine to generate isopentylamine, an aliphatic monoamine, and trace amine. To identify the function of LDC-1-produced isopentylamine and assuming that it may be involved in cellular metabolism, we incubated opossum kidney cells (OKC) with 1 mM isopentylamine and measured the cellular oxygen consumption rate (OCR). Isopentylamine significantly increases cellular OCR (Fig. 6D). To identify the role of ZBTB16, we established an inducible stably transduced *Zbtb16* overexpressing cell line generated from OKC (Fig. 6E). It was demonstrated that ZBTB16 plays a role in cellular bioenergetics and in autophagy by mediating the proteasomal degradation of autophagic protein Atg14L, and in the pathogenesis of metabolic diseases [17, 18]. We, therefore, measured the proliferation, cellular respiration, and transcription levels of genes that were differentially expressed in the results of RNA sequencing. No difference was encountered (Supplemental Fig. 6B–D). Cellular autophagy was determined by western blotting of microtubule-associated protein-1 light-chain (LC3) and p62 levels. LC3II levels were significantly reduced upon ZBTB16 overexpression and p62 levels were significantly higher suggesting a role in inhibiting autophagy (Fig. 6F, G). To elucidate whether the decrease of LC3II was due to reduced autophagosome formation or to an increase of autophagic degradation, control, and ZBTB16 overexpressing cells were treated with bafilomycin A1, an inhibitor of autophagosome-lysosome fusion. LC3II accumulated in both control and in ZBTB16 overexpressing OKC to a similar extent (Fig. 6H), indicating a diminution of autophagosome formation rather than enhancement of autophagic flux.

## Discussion

**Fig. 7:**
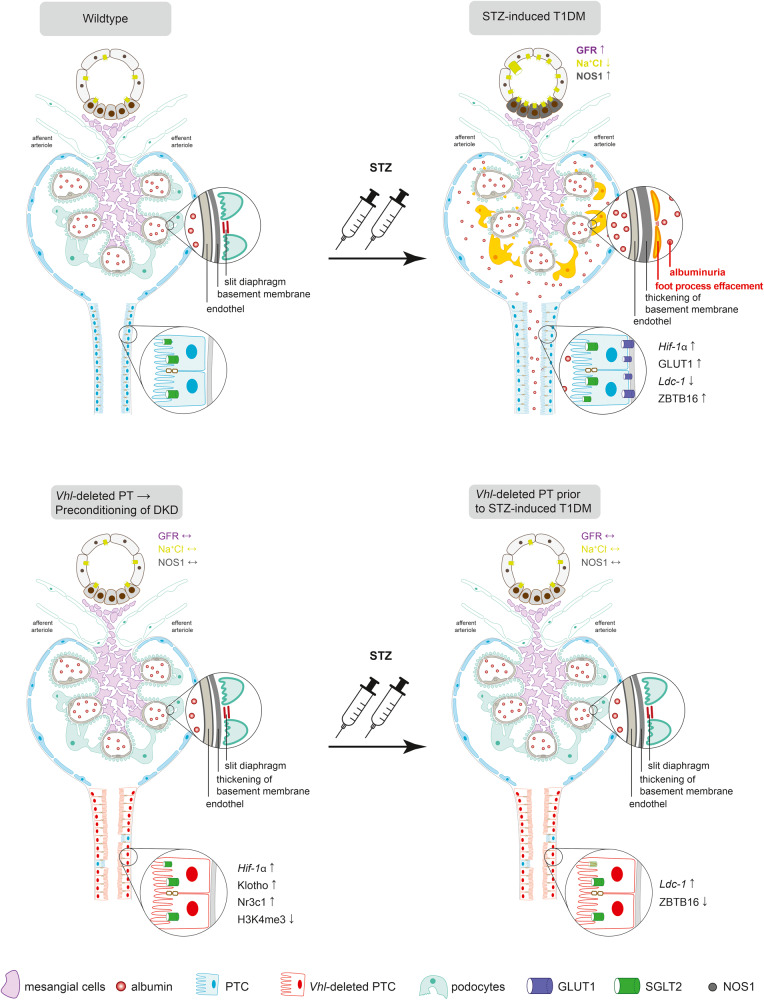
Proximal tubular *Vhl* deletion preconditions against diabetic kidney disease. Scheme illustrating glomerular and tubular alterations including glomerular hyperfiltration, proteinuria, foot process effacement, basement membrane thickening, tubular transport, PT cell growth, and tubular HIF-1α stabilization in DKD. *Vhl*-induced deletion preconditions against DKD mediated by high long-term HIF-1α stabilization, Klotho and glucocorticoid receptor (Nr3c1) expression, and low H3K4me3 modification and thereby normalizing GFR, tubular transport, metabolism, gene expression profile, and ameliorating kidney morphology. T1DM type 1 diabetes mellitus, GFR glomerular filtration rate, PT proximal tubule, PTC proximal tubule cell.

Genetic PT-specific *Vhl* deletion-induced alterations in gene expression profile and renal morphology resembling that of STZ-induced type 1 DM albeit to a lesser extent. *Vhl* deletion prior to STZ-induced diabetes prevented glomerular hyperfiltration and proteinuria, and helped to preserve renal function and morphology, with normalized metabolism and total gene expression profile by histone modification and chromatin remodeling. As a whole, PT-specific *Vhl* deletion preconditions against DKD, as summarized in Fig. 7.

### Tubulocentric view of DKD and tubular alterations

There is emerging evidence of PT involvement in the initiation and contribution to the early pathogenesis of DKD. Glomerular hyperfiltration is a hallmark of early DKD and predominantly results from hyperglycemia enhancing the filtration of glucose. The PT consequently increases its sodium and glucose reabsorption rates, thereby limiting the delivery of sodium and chloride to the macula densa. This resultant inhibition of the TGF further causes afferent arteriolar vasorelaxation, thereby increasing renal blood flow and hyperfiltration [19]. SGLT1 in the macula densa senses glucose to regulate the production of nitric oxide (NO) by NO synthase 1 (NOS1) when the sodium-glucose uptake is saturated. In addition, increasing amounts of glucose delivered to the macula densa stimulate NOS1-mediated NO production to further augment the GFR [20]. Thus, in the early stages of diabetes, the tubular hypothesis proposes that the TGF is the main controller of GFR. In our study, STZ-induced DKD demonstrated augmented NHE3 and NKCC2 leading to reduced sodium delivery to the macula densa, which further enhances the GFR. In addition, NOS1 expression was increased suggesting glucose transporter saturation and a further rise in GFR. The augmented NKCC2 expression, as shown in previous publications using STZ-induced diabetic animal models [21, 22], occurred by increasing *Slc12a1* transcripts and was independent of vasopressin-mediated phosphorylation. PT *Vhl* deletion prior to STZ treatment was unexpectedly not associated with increased expression of sodium and glucose transporters and NOS1, which may explain the prevention of glomerular hyperfiltration in VHL^ΔPT^/STZ.

To identify HIF-1α driven processes in DKD, we grouped commonly altered genes obtained from our RNA sequencing results (Fig. 3). Commonly altered genes include genes for cell polarity such as *Notch1*, *Btbd7,* and *Fryl*, which are involved in kidney development. For *Notch1*, increased expression was found in db/db type 2 diabetic mice and in human biopsies, in association with inflammation [23, 24] proteinuria, and basement membrane thickening in DKD [24]. Interestingly, basement membrane thickening was found in our morphological analysis in VHL^ΔPT^, con/STZ, and VHL^ΔPT^/STZ, supporting the results of RNA sequencing analysis. Elevated levels of *Hif1α*, *Nox4, Rictor,* and *Nfat5* have been involved in hypoxic, oxidative, cell, or osmotic stress and disease progression in mouse and human kidney samples with DKD. [11, 25–28]. In addition, higher mRNA levels of several transporters and channels were observed including *Slc9a3* (coding for NHE3) and *Slc12a1* (coding for NKCC2), similar to the results at the protein level (Fig. 2 and Supplemental Fig. 2), thus confirming RNA sequencing data. Genes important for metabolic adaptation to diabetes include the transcription factor *Clock*, playing a role in PT gluconeogenesis and consequent serum glucose concentration in STZ-induced type 1 DM [29], *Kdm1*, an epigenetic regulator for salt-sensitive hypertension, and the kinases *Pik3c2a*, *Pkn2* [30]. The diabetic tubule further possesses alterations in genes affecting PT endocytosis, such as *Cltc* (clathrin) and *Eea1* (early endosomal antigen) congruent with the higher number of invaginations per PT surface, which is in agreement with increased PT reabsorption rates in DKD [31]. Morphologically, increased tubular height and basement membrane thickening have been observed in PT upon *Vhl* deletion or STZ treatment representing a feature of HIF-1α stabilization. In addition, as shown in earlier studies from our group, tubular *Vhl* deletion was shown to increase epithelial cell proliferation [32], known to occur in PT in early DKD [20].

### Cellular HIF-1α stabilization

We show that PT *Vhl* deletion leads to consequent HIF-1α stabilization as judged by nuclear HIF-1α protein expression levels and from RNA sequencing results (Figs. 1D and 3C). HIF-1α stabilization occurred in both VHL^ΔPT^ and con/STZ, as nicely demonstrated earlier [11]. In VHL^ΔPT^/STZ, we observed a significant reduction of nuclear HIF-1α protein expression levels compared to VHL^ΔPT^ albeit PT HIF-1α downstream target expression. This may be due to the preconditioning found in that group, affecting many of the genes altered including *Hif-1α*. In humans, mutations in the *Vhl* gene are associated with degradation of cilia and renal cell carcinoma formation [33]. In mice, no obvious alterations in kidney and liver morphology were detected up to 9 months after *Vhl* deletion [34], suggesting that in mice, an additional event may be necessary.

### Tubuloglomerular crosstalk

The diabetic PT is suggested to be the primary pathological event in DKD [3, 35], and was shown to contribute to podocyte or mesangial cell injury in a paracrine fashion [3, 36]. In our model, we have observed that PT *Vhl* deletion-induced alterations in glomerular genes related to morphology including the respective morphological changes such as angiogenesis and glomerular basement membrane (GBM) thickening. As reported previously from our group, tubular *Vhl* deletion-induced glomerular angiogenesis [32], which we re-observed with *Vhl* deletion in PT S1 and S2 segments. Glomerular angiogenesis is a hallmark of DKD. Among the genes, commonly regulated in *Vhl*-deleted PT and STZ-induced type 1 diabetes, we found f. e. *Ptprb, Ogt, Ctss*, known to be involved in vascular changes during DM [14, 35, 37]. Thickening of the glomerular basement membrane is in agreement with higher gene expression for *Nid2*, *Lamc1*, *Col4a3,* and *Col4a5*, coding for components of the basement membrane suggesting a structural remodeling characteristic for DKD [38]. Our PT *Vhl-*deleted mice imply that PT HIF-1α-stabilization participates in the diabetic glomerular phenotype. *Vhl* deletion prior to STZ treatment prevented podocyte loss with concomitant foot process effacement, which is in agreement with the prevention of albuminuria as shown previously [39]. Furthermore, high VEGF levels in podocytes and renal tubules were attributed to increased glomerular size and Kimmelstiel-Wilson-like nodular glomerulosclerosis [15, 16]. Our results revealed no difference in glomerular VEGF levels in our mouse model despite higher glomerular size in STZ-treated groups, therefore we assume that higher tubular VEGF expression may have affected glomerular growth. The resulting lower VEGF backflow in con/STZ, and in renal biopsies with mild and moderate DM may most probably be due to increased matrix accumulation interfering with the VEGF backflow. Reduced VEGF levels in severe DM may be due to podocyte damage and loss. As an underlying mechanism we found in comparison to other renal epithelial cells, that podocytes are more susceptible to the diabetic setting (hypoxia and high glucose) reacting with a decline in VEGF production which was shown earlier to be a result of podocyte damage [40].

### Proximal tubular HIF-1α stabilization preconditions the kidney against diabetes-induced changes

Our data revealed that glomerular and tubular morphological changes including renal cell function at mRNA level are similar between PT *Vhl* deletion and STZ-treated mice, albeit to a lesser extent. In addition, *Vhl* deletion induced prior DM ameliorated glomerular and tubular morphology and normalized gene expression profile suggesting that PT HIF-1α stabilization prior to DM preconditions against DKD. Our observations go in line with recent publications: administration of a HIF-1 inhibitor to the diabetic mouse model OVE26 with established DKD prevented mesangial matrix expansion, glomerulosclerosis, and albuminuria [41]. In the same direction, transgenic mice overexpressing VEGF along the tubule resulted in enlarged glomeruli with mesangial proliferation and mesangial nodules similar to DKD [16]. However, stabilizing HIF-1α before the onset of type 1 or type 2 diabetes normalized GFR, reduced oxygen consumption and proteinuria [42], ameliorated glomerular and endothelial damage [39], and counteracted renal metabolic alterations [43]. As a limitation of our study having used the SGLT2-cre mouse model, we cannot rule out developmental effects on PT function, which may have affected DKD establishment. In agreement, our results provide new insight that HIF-1α may also precondition the kidney against DKD. Mechanistically, we showed that long-term HIF-1α stabilization leads to a strong reduction in histone methylation as shown by H3K4me3 levels and thereby resetting all genes altered towards the control level despite type 1 DM. Numerous studies have already revealed that histone methylation plays an essential role in DKD progression, especially H3K4me3 with its conserved distribution on gene promoters [44, 45]. High levels of H3K4me3 were associated with inflammatory and oxidative stress, progressive proteinuria through podocyte injury, and fibrosis [44–46], thus participating in DKD progression. Hypoxia was shown to rapidly increase histone methylation and chromatin reprogramming at various sites [47]. We can now show, that long-term HIF-1α stabilization leads to a strong reduction in H3K4me3 levels and thereby preventing hallmarks of DKD.

Among the genes, which were significantly different between con/STZ and preconditioned VHL^ΔPT^/STZ were *Slc12a1*, *Zbtb16,* and *Ldc-1*. We could confirm higher NKCC2 protein levels (coded by *Slc12a1)* in the STZ-induced type 1 diabetic model. ZBTB16 belongs to the promyelocytic leukemia zinc finger family and is a multifaceted signaling hub for a number of cellular processes and was recently presented to be involved in the pathogenesis of metabolic diseases by regulation of cell bioenergetics [17, 18]. However, overexpressing ZBTB16 in OKC revealed a role as a negative regulator of autophagy as demonstrated previously [48, 49]. *Ldc-1*, coding for leucine-decarboxylase 1, catalyzes isopentylamine [50]. We found that isopentylamine stimulated PT cellular oxygen consumption and therefore cell metabolism. The higher and therefore normalized *Ldc-1* levels in VHL^ΔPT^/STZ compared to con/STZ may ameliorate cellular bioenergetics and help the PT cells to adjust to the augmented cellular energy demand in type 1 diabetes.

Current prevention of DKD includes the optimization of cardiovascular risk factors, including hyperglycemia, hyperlipidemia, obesity, smoking, and hypertension. Interfering with the glomerular hemodynamics by the means of ACE-inhibitors/sartans or SGLT2 inhibitors attenuates the hyperfiltration and reduces proteinuria, thereby slowing down the progression of DKD [51]. On the basis of our observations in a mouse model of STZ-induced type 1 DM, one may speculate that HIF-1α stabilizers may similarly help to prevent DKD development in patients with type 1 DM.

In summary, genetic PT *Vhl* deletion demonstrated similar morphological changes and gene expression patterns similar to STZ-induced type 1 diabetes. PT *Vhl* deletion prior to STZ-induced type 1 diabetes prevented glomerular hyperfiltration and proteinuria, normalized gene expression pattern by reduction of trimethylation of histone 3 (H4K4me3) and preserved renal morphology, thereby preconditioning the kidney against DKD (Fig. 7).

## Material and methods

### Animal experimentation

All animal experiments were conducted according to the National Institutes of Health guide for the care and use of laboratory animals, the Swiss law for the welfare of animals, ARRIVE guidelines, and they were approved by the Cantonal Veterinary Office (Canton of Fribourg, Switzerland) (FR25959, 2014_55_FR). All protocols were reviewed by the University’s Animal Welfare and Ethics Review Board before experimentation. Mice were housed in an SPF facility with free access to chow and water on a 12-h day-night cycle. Breeding and genotyping were done according to standard procedures. *Vhl*^fl/fl^ mice have been described previously [52] and were crossed to SGLT2^Cre^ mice (Tg(Slc5a2-cre)1Tauc [53]), European mouse mutant archive). After prior POWER analysis, littermates were used in a randomized fashion and were on a C57BL/6/FVB mixed background. DM was induced in male *Vhl*^fl/fl^ (termed control) and *Vhl*^fl/fl^/SGLT2^Cre^ (termed VHL^ΔPT^) mice at 12 weeks of age. After overnight fasting, control and VHL^ΔPT^ mice were injected with streptozotocin STZ (100 mg/kg) *i.p*. for two consecutive days. STZ (SigmaAldrich, Munich Germany) was prepared in 0.1 mol/L sodium citrate buffer (pH 4.5) immediately before injection. Control animals received citrate buffer alone. The onset and progression of diabetes were evaluated by measuring blood glucose levels from blood samples obtained after 4 h fasting using Accu-Chek test stripes (Roche Applied Science) once a week in the subsequent weeks. Mice with blood glucose levels higher than 300 mg/dl after 1-week post STZ exposure till the end of the experiment were considered diabetic.

Human kidney biopsies were retrieved from the archive of the Institute of Pathology at the University of Mainz, Germany. Samples from patients with biopsy-proven diabetic nephropathy were graded according to the severity of the glomerular changes as mild, moderate, and severe. Biopsies from non-diabetic patients with the diagnoses of mild tubulointerstitial damage and/or inflammation and lacking glomerular alterations served as controls. The use of archive material for scientific purposes was approved by the ethics committee under the number 837.360.16.

### Cell culture

If not stated otherwise, all cell lines were cultured in a cell-specific growth medium (medium composition see below) and incubated at 37 °C in a 5% CO_2_ and 95% air atmosphere. Opossum kidney (OK) cells were cultured in poly-d-lysine-coated cell culture flasks. The growth medium of OK cells was composed of 1:1 mixture of Dulbecco’s modified Eagles medium (DMEM; PAN Biotech; P04-03590) and Ham’s F-12 (PAN Biotech; P04-15500) supplemented with 10% fetal bovine serum, 2 mM glutamine, 100 U/mL penicillin, 100 µg/mL streptomycin, 3,0 g/L Na_2_CO_3_ and 5 mM HEPES. To generate OK cell line overexpressing ZBTB16 (PLZF) we used lentiviral supernatant produced by Dr. Emmanuel Di Valentin (University of Liège, GIGA-Viral Vectors Platform) using ViraSafe™ Lentiviral Packaging System, Pantropic (VPK-206) with TET-On 3G system. Briefly, HEK 293T cells were transient transfected with plasmids encoding for the components of the virion and the transgene sequences for human PLZF (TetO-FUW-PLZF, gift from Rudolf Jaenisch, Addgene plasmid # 61543 [54], transactivator protein (pLV EF1a TET3G) and GFP (pLV TREG EmGFP). Lentivirus was harvested, concentrated and purified, and applied to target cells in two sequential transductions. We first established a cell line only expressing transactivator protein (referred to as “inducer”) by incubating OK cells with EF1a TET3G (3.18E + 08 TU/mL) in the presence of 6 µg/mL of diethylaminoethyl (DEAE)-dextran followed by selection with 100 µg/mL hygromycin. The “inducer” cell line was used for second transduction with TetOn hPLZF (referred to as “ZBTB16”) and TREG EmGFP (referred to as “GFP”) serving as fluorescent transduction control. “ZBTB16” and “GFP” cell lines were cultured in a growth medium supplemented with tetracycline-free FBS and 100 µg/mL hygromycin. Autophagy flux was analyzed using bafilomycin 200 nM (Cayman Chemicals), 2 h prior to cell lysis of OK cells.

Conditionally immortalized murine podocytes (gift from Prof. N. Endlich, Greifswald University Medicine, Germany) and murine cortical collecting duct mpkCCD_c14_ cells (gift of Prof. Kortenoeven, University of Southern Denmark, Denmark) were used for hypoxia and high-glucose treatment. Podocytes were handled as described by Endlich et al. [55]. Briefly, podocytes were cultured in RPMI 1640 medium (Sigma Aldrich) supplemented with 10% fetal bovine serum (FBS; PAN), 100 U/mL penicillin, and 0.1 mg/mL streptomycin (Sigma Aldrich). For propagation, podocytes were cultured at 33 °C (permissive conditions). To induce differentiation, podocytes were maintained at 37 °C (nonpermissive conditions) for at least 10 days. The murine cortical collecting duct mpkCCD_c14_ cells were cultured in a 1:1 mixture of Dulbecco’s modified Eagles medium (DMEM; PAN Biotech; P04-03590) and Ham’s F-12 (PAN Biotech; P04-15500) supplemented with 60 nM Na^+^ selenate, 5 μg/mL transferrin, 50 nM dexamethasone, 1 nM triiodothyronine, 10 ng/mL epidermal growth factor, 5 μg/mL insulin, 2% fetal bovine serum, and 100 U/mL penicillin, 100 µg/mL streptomycin. For hypoxia and high-glucose treatment, differentiated podocytes and mpkCCD_c14_ were incubated in a starvation medium (FBS- and glucose-free) overnight under normoxic conditions (21% O_2_, 5% CO_2_ at 37 °C). Then, cells were exposed to high glucose at 25 mM (Merck) and to a 3% oxygen environment at 37 °C, 5% CO_2_ using a hypoxic chamber (Memmert, AtmoCONTROL) for 6 h or 24 h. Samples were collected for qRT-PCR analyses. OKC, mpkCCD_cl14_ cells, and immortalized podocytes were mycoplasma-free and are tested regularly once a month.

### Determination of oxygen consumption

To enhance cell differentiation to a more physiological phenotype OK cells were seeded in a 96-well plate at a density of 40,000 cells/well in a growth medium and cultured under standard conditions. After 24 h, cells were cultured on an orbital shaker at 1 Hz shear conditions. After another 24 h, cells were washed with PBS, and the medium was replaced by 70 µL low glucose medium (DMEM [GIBCO; A1443001] including 1 g/L glucose, 3,7 g/L sodium bicarbonate, 4 mM glutamine, 100 U/mL penicillin and 100 µg/mL streptomycin) supplemented without or with 1 µg/mL doxycycline. After another 48 h, cells were washed with PBS and cultured in 70 µL no glucose medium (DMEM [GIBCO; A1443001] including 2,2 g/L sodium bicarbonate, 2 mM glutamine, 100 U/mL penicillin and 100 µg/mL streptomycin) for 24 h. After this treatment, the oxygen consumption rate was measured using the Extracellular Oxygen Consumption Assay (Abcam ab197243) according to the manufacturer’s recommendations. To test a potential effect of isopentylamine/isoamylamine (Sigma, cat.nr.126810) on oxygen consumption rate (OCR) using OK cells, isopentylamine (1 mmol/L as established in Ferrero et al. [56])) was added to the corresponding wells prior to measurement. Experiments were performed in duplicate and repeated 3 times.

### Measurement of glomerular filtration rate in conscious mice

Glomerular filtration rate (GFR) was studied ten weeks after diabetes induction. Mice received a bolus injection of FITC-sinistrin (2 µg/g bw; MediBeacon, Mannheim Germany) i.v. followed by blood sampling at 3, 5, 7, 10, 15, 35, 56, and 75 min after injection according to Rieg et al. [57]. Sample fluorescence intensities were measured using a plate reader (GENios, TECAN, Austria), and a two-phase exponential decay curve fitting was performed using GraphPadPrism software to calculate GFR.

### Fluid intake, blood, and urine collection/analysis

Mice were placed for 24 h in metabolic cages for urine collection. Retroorbital blood sampling was performed subsequently. Blood and urine samples were analyzed at the Fribourg Cantonal Hospital (Laboratoire HFR) using an ion-selective electrode indirect method for the detection of Na^+^, K^+^, Cl^-^, Cat. No. 0588392001 (ISE indirect Na^+^, K^+^, C^-^ for Gen.2, Roche, Basel Switzerland); quantitative determination of creatinine, kit CREJ2- Cat. No. 04810716 190 (Creatinine Jaffé Gen.2, Roche, Basel Switzerland) and; quantitative determination of urinary protein using TPUC3, Cat. No. 03333825 190 (Total Protein Urine/CSF Gen.3, Roche, Basel Switzerland) on instrument Cobas 6000 (Roche, Basel, Switzerland). Analysis was performed in a blinded fashion.

### Fixation and tissue processing for immunohistochemistry and immunoblotting

Mice were anesthetized by intraperitoneal injection of ketamine/xylazine and kidneys were removed and shock-frozen for biochemical evaluation or perfused retrogradely through the aorta by using 4% PFA in PBS. Perfusion-fixed specimens were post-processed for cryo-, paraffin-, and epon-embedding for further histochemical, light, and electron microscopy analysis. For immunoblotting, membrane and nuclear fractions were prepared from shock-frozen renal cortices. Membrane fractions were prepared using 0.25 mol/L sucrose buffer containing triethanolamine (0.13%), proteases inhibitors, and phosphatases inhibitors (Roche) and processed as described [58]. Soluble nuclear and insoluble nuclear protein fractions were prepared using the Nuclear Extract Kit (Abcam, ab219177) according to the manufacturer’s recommendations. Protein content was determined using the Pierce™ BCA Protein Assay Kit (Thermo Fisher Scientific, Waltham, USA), and protein amounts were adjusted based on coomassie staining.

### RNA isolation and RNA sequencing

RNA was isolated from frozen kidney cortices according to the manufacturer´s instruction (NucleoSpin RNA/protein, Machery & Nagel). 2 µg RNA was reverse transcribed into cDNA in the presence of M-MLV reverse transcriptase (Promega), dNTP and random hexamer primer. RNA isolated from renal cortices of 2 mice were pooled for one RNA sequencing measurement with *n* = 6 mice for control, *n* = 4 mice for VHL^ΔPT^, *n* = 4 mice for con/STZ, and *n* = 4 mice for VHL^ΔPT^/STZ. The RNA quality expressed as RIN^e^ used for RNA sequencing analysis was 7.5 ± 0.4 (mean ± SEM).

#### RNA-Seq sequence details

Paired-end sequencing libraries were constructed for replicates of each genotype from the total RNA isolated, employing the True Seq stranded mRNA Poly ATrue Seq stranded mRNA Poly A library kit. Subsequent sequencing was performed on the Illumina HiSeq3000 (1 × 50 bp) using standard protocols.

#### RNA-Seq pipeline

We used our in-house RNA-Seq pipeline to map and align the sequenced data (https://github.com/nf-core/rnaseq). The workflow processed the raw data from the sequencer with FastQC v0.11 [59] and Trimgalore v0.4 [60]. aligned the reads with STAR v2.5.2b [61] and generated gene counts with feature counts v1.5.2 [62] and StringTie v1.3.3b [63]. Quality control was assessed throughout with RSeqQC [64], dupRadar [65], Preseq [66], and MultiQC v1.4 [67]. As reference genomes, we used GRCm38 *Mus musculus* genome (Genome Reference Consortium Mouse Build 38 and GenBank Assembly ID: GCA_000001635.8), with the total of reads per sample aligning on an average of 83.56%.

#### RNA-Seq analysis

Differentially expressed genes were identified by comparing the expression profiles of the different genotypes. Statistical analysis was performed using R v3.6.3.To identify differentially expressed genes between patients and time points, we used DESeq2 R package v1.26.0 [68]. DESeq2 was used for executing pairwise comparisons between genotypes. This statistical tool is based on a negative binomial distribution model with dispersion trend smoothing and was also used to determine the normalized read counts per sample by estimating size factors to control for library size, followed by a log2 transformation of the raw count data using DESeq2. Gene ontology analysis was performed employing a two-sided Fisher’s exact test as described before [69]. The corresponding biological processes were obtained from the Gene Ontology Consortium (www.geneontology.org).

### Quantitative RT-PCR (qRT-PCR)

Total RNA was extracted from cultured opossum kidney cells (OKC), podocytes, and mpkCCD_c14_ cells using NucleoSpin RNA purification kit (Macherey-Nagel, Düren Germany) according to the manufacturer´s protocol. For cDNA synthesis, reverse transcription was performed using random hexamer primers (0.2 µg/µL; Thermo Scientific, Munich Germany) and M-MLV RT RNase (Promega, Walldorf Germany). Quantitative real-time PCR was performed using an ABI 7500 Fast Real-Time PCR System (Applied Biosystems, Dreieich Germany) with either qPCR MasterMix Plus (Eurogentec) and TaqMan gene expression assays ID Mm00437304_m1 (Vegfa FAM) and Mm00446973_m1 (Tbp VIC) or HOT FIREPol® EvaGreen® qPCR Mix Plus (Solis BioDyne, Tartu Estonia) for genes listed in Table S2. The relative abundance of *Vegfa* mRNA in podocytes and mpkCCD_c14_ cells was normalized to that of *Tbp* using the comparative cycle threshold method (2^−ΔΔCT^); genes listed in Table S2 were normalized against the housekeeping gene *Gapdh*.

### RNA in situ hybridization

For BaseScope™ and RNAScope^R^ analysis, 5 µm paraffin sections were stained with BaseScope™ Detection Reagent Kit v2-RED (cat. no. 323910, Bio-Techne, Abingdon, UK) using a 3-ZZ mouse *Vhl* probe directed against Exon 1 (cat. no. 715931, accession no. NM_009507) or RNAScope^R^ 2.5. HD Assay-RED (cat. no. 322350, Bio-Techne, Abingdon, UK) using a mouse *Ldc-1* probe (cat. no. 864121), respectively. Scopes were carried out according to the manufacturer’s instructions. To verify mRNA quality a probe against the housekeeping gene peptidylprolyl isomerase B (cat. no.701071, accession no. NM_011149.2) served as positive control. As negative control served a probe against the bacterial gene DapB (cat. no. 701011, accession no. EF191515). Afterward, sections were counterstained with anti-SGLT2 (kindly provided by H. Koepsell, Anatomy Würzburg Germany) to mark S1/2 proximal tubular segments or LRP2/megalin to mark total proximal tubule and DAPI for nuclei. Multicolor images from renal cortices were taken as Z-stacks using Leica Axiovert microscope and the number of VHL-positive cells per glomeruli or in SGLT2-positive proximal tubules compared to all SGLT2-positive proximal tubule cells was determined. For *Ldc-1* probe multicolor images from kidney cortices were taken using Abberior Facility Line multilaser confocal scanning microscope and *Ldc-1*-positive cells in LRP2/megalin-positive proximal tubules were analyzed.

### Sodium dodecyl-sulfate polyacrylamide gel electrophoresis (SDS-PAGE) and immunoblotting

Proteins were solubilized and SDS gel electrophoresis was performed on 10% polyacrylamide gels. After the electrophoretic transfer of the proteins to nitrocellulose membranes, equity in protein loading and blotting was verified by membrane staining using 0.1% Ponceau red. Membranes were probed with primary antibodies (s. list) and then exposed to HRP-conjugated secondary antibodies (Dianova, Hamburg, Germany). Immunoreactive bands were detected by chemiluminescence using Immobilon Western HRP substrate (Millipore, Darmstadt, Germany) in combination with the chemiluminescence imaging system Fusion SL (Peqlab, Erlangen, Germany) and further analyzed using ImageJ software. The resulting values are presented in the percent of control values obtained from the control group. Immunoreactive bands were normalized to the corresponding housekeeping proteins as indicated in the figures. Uncropped immunoblots are shown in Supplemental Fig. 7.

### Immunohistochemistry/immunocytochemistry

Paraffin sections were antigen-retrieved by high-pressure cooking in 0.1 M citrate buffer pH=6.0, followed by blocking endogenous peroxidase with 3% H_2_O_2_ in 100% methanol and by a blocking step using 5% skim milk/PBS. Sections were incubated with the respective primary antibody overnight followed by the suitable HRP- or fluorescence-coupled secondary antibody (Dianova). Signals were generated using 3,3’-diaminobenzidine for HRP staining. Specimens were analyzed using the Leica Axiovert microscope or STED microscope (FACILITY Line, Abberior).

Five-micrometer cryo sections were antigen-retrieved using 0.5% tritonX-100/PBS, blocked with 5% skim milk/PBS, incubated overnight at 4 °C with either acetylated tubulin, nNOS/NOS1, klotho, or glucocorticoid receptor followed by incubation with a suitable fluorochrome-coupled secondary antibody (Dianova). Nuclei were counterstained with DAPI (4′,6-diamidino-2-phenylindole, 100 ng/mL).

Cells grown on glass coverslips were fixed with 4% paraformaldehyde (PFA)/PBS for 10 min, permeabilized with 0.5% tritonX-100/PBS for 30 min, and blocked with 5% skim milk/PBS for 1 h. For detection of cell borders, cells were stained with ZO-1 followed by incubation with anti-ZBTB16 (Sigma; cat.no. HPA001499) and suitable fluorochrome-coupled secondary antibody (Dianova). Samples were analyzed using a STED microscope (FACILITY Line, Abberior).

### Antibodies

The following antibodies were used: rabbit anti-SGLT2 (generously provided by H. Koepsell), guinea pig anti-SGLT2 (generated by Pineda), guinea pig anti-NKCC2 (gift. S. Bachmann), rabbit anti-pNKCC2 (gift. S. Bachmann), rabbit anti-GLUT1 (Stressmarq, Victoria Canada), rabbit anti-NHE3 (NBP1-8257 Novus, Stressmarq), goat anti-VEGF (R & D Systems), rat anti-Meca32 (PV-1, Abcam), rabbit anti-ZBTB16 (LSBio; LS-C680061 and Sigma; HPA001499), rabbit anti-Zbtb16 (Sigma Aldrich), rabbit anti-Klotho (Abcam, ab181373), rabbit anti-glucocorticoid-receptor (Gene Tex; GTX101120), mouse anti-GAPDH (Santa Cruz, sc-32233), rabbit anti-Histone 3 (Proteintech; 17168-1-AP), mouse anti-β-actin (Santa Cruz, sc-4777), rat anti-ZO-1 (Millipore, R40.76; MABT11), rabbit anti-nNOS (Cayman Chemical; cat.no.160870-1), guinea pig anti-LRP2 [70], Alexa-647-phalloidin (Molecular Probes; A22287), rabbit anti-PCNA (Proteintech; cat.no 60097-1-Ig), rabbit anti-GFP (Cell Signaling; cat.no. 2037), rabbit anti-LC3A/B (Abcam; ab128025), rabbit anti-SQSTM1/p62 (St John’s Laboratory; STJ195471), rabbit anti-HIF-1α (Novus; NB100-479), mouse anti-acetylated tubulin (Sigma Aldrich; clone 6-11B-1), rabbit anti-VEGF-A (Abcam, ab52917), mouse anti-flotillin-1 (BD Transduction; cat.no. 610820), and rabbit anti-H3K4me3 (Abcam, ab8580).

### Morphometry and glomerular and tubulointerstitial injury score

Morphometry. Images of glomeruli from PAS-stained paraffin sections and toluidine blue-stained semi-thin sections of each animal per group were taken using a light microscope (Axiovert, Zeiss) and the size of glomerular tuft, Bowmans capsule, and capillary area was determined using Fiji ImageJ. For measurement of Bowman’s capsule and glomerular tuft area, only glomeruli with visible extracellular mesangium and macula densa were analyzed. Ultra-thin sections were stained with uranyl acetate and lead citrate and transmission electron microscopy (TEM) was used to evaluate the morphology of the glomerular filtration barrier and the early proximal tubule epithelial cells (PTC) of the kidney. Only tangential cut glomerular filtration barrier and PTC were analyzed. Images from ultra-thin sections were taken using TEM (JEM 1400 plus, JOEL) and TemCam F416 (TVIPS). Then, glomerular and tubular basement membrane thickness, podocyte foot processes per glomerular basement membrane, tubular epithelial height, microvilli length, and invaginations per cell surface were determined using Fiji ImageJ. PT albumin uptake was determined by counting albumin-positive vesicles per PT nuclei on immunohistochemically stained paraffin sections. Glomerular and tubulointerstitial injury score. Periodic acid-Schiff (PAS) stained 5 µm paraffin sections were studied to evaluate the degree and extent of glomerular injury. Signs of glomerular damage comprised thickening of the glomerular basement membrane, mesangial expansion, and podocyte hypertrophy. A semi-quantitative score was applied and the severity of damage for each glomerulus was graded from 0 to 4 as follows: 0 represents no lesion, 1 represents glomerular basement membrane thickening and mesangial matrix expansion, 2 represents additional podocyte hypertrophy, 3 represents additional podocyte loss, and 4 represent additional nodular mesangial lesions and mesangiolysis. A whole-kidney average score was obtained by averaging scores from all glomeruli in one section. For the tubulointerstitial injury characterization tubular dilation and atrophy, necrosis, proteinaceous casts, and fibrosis were assessed per 20x images taken from kidney cortices and severity was graded from 0 to 4 as follows: 0 represents no lesion, 1 injury <25% of the image, while 2, 3, and 4 represent injury of 25 to 50%, 50 to 75%, and >75% of the image, respectively. Circa 20–30 images per kidney per animal were determined. All analyses were performed in an observer-blinded fashion.

### Statistical analysis

Statistical analysis was performed using GraphPad Prism 7.0 (GraphPad Prism). Data are provided as arithmetic means ± SEM with *n* representing the number of used mice/ samples. To test statistical significance, one-way ANOVA (Kruskal–Wallis test) followed by Dunn´s test was performed for comparing three or four groups; student’s *t*-test (nonparametric Mann–Whitney-*U*-test) was used for the comparison of two groups in cell culture experiments (GraphPad 7.0). *P < 0.05, **P < 0.01, ***P < 0.001 was considered statistically significant.

## Supplementary information

Supplemental Material

Supplemental Table 1

uncropped western blot data

## Data Availability

All data generated or analyzed during this study are included in the published article and supplementary material. Raw data and processed data from RNA sequencing analysis are uploaded on GEO (https://www.ncbi.nlm.nih.gov/geo/; accession number: GSE210401). No applicable resources were generated or analyzed during the current study. Supplementary information is available at *Cell Death & Disease*’s website. Additional data are available from the corresponding author on reasonable request.
