# Supplementary material for: Targeted deletion of von-Hippel-Lindau in the proximal tubule conditions the kidney against early diabetic kidney disease
Source: Cell Death Dis. 2023 Aug 26;14(8):562. doi: 10.1038/s41419-023-06074-7 (PMC10457389; doi:10.1038/s41419-023-06074-7)
Supplement: Supplementary file 1 — Supplemental Material [file 41419_2023_6074_MOESM1_ESM.docx]

**Supplemental Figures**


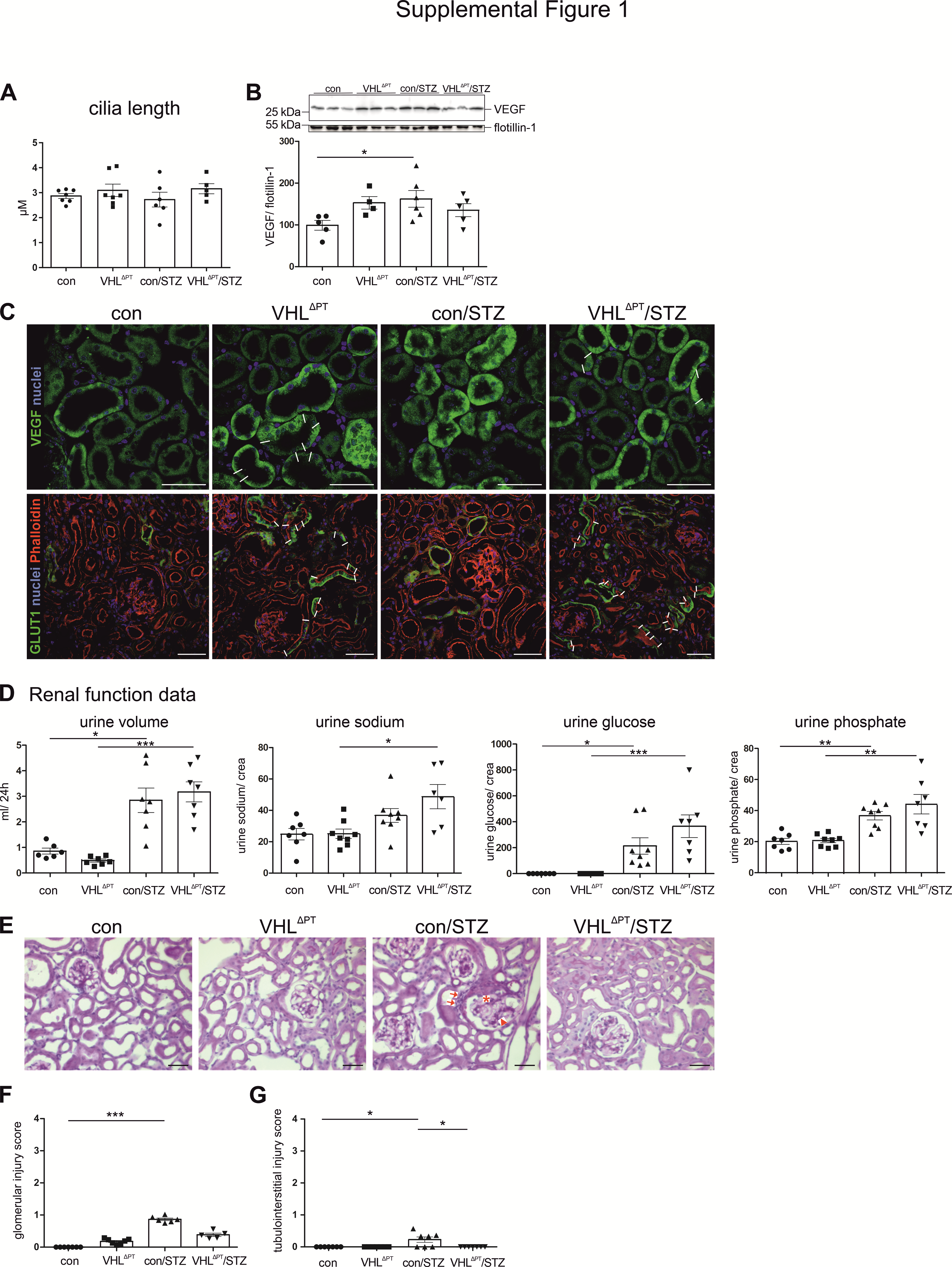


**Supplemental Figure 1. Cilia length, expression of VEGF and GLUT-1, renal function data and renal morphology.** (**A**) Cilia length in µm. Arithmetic means ± SEM of n = 4-7 per group. (**B**) Western blots of renal VEGF abundance and densitometric evaluation of control, VHL^∆PT^, con/STZ and VHL^∆PT^/STZ with flotillin-1 as reference. Arithmetic means ± SEM of n = 4-5 per group; **P* < 0.05. (**C**) Representative images of immunohistochemical staining of VEGF and GLUT-1 obtained from control, VHL^∆PT^, con/STZ and VHL^∆PT^/STZ. Scale bar = 50 µm. (**D**) Renal function data: urine volume, urine sodium, urine glucose and urine phosphate of control, VHL^∆PT^, con/STZ and VHL^∆PT^/STZ. Arithmetic means ± SEM of n = 5-7 per group; **P* < 0.05, ***P* < 0.01, ****P* < 0.001. (**E**) Representative images of PAS stained renal cortices of control, VHL^∆PT^, con/STZ and VHL^∆PT^/STZ. Scale bar = 50 µm. Note, the matrix expansion (asterisk), glomerular basement membrane thickening (arrow head) and fibrotic area (arrow). (**F** and **G**) Semi-quantitative evaluation of glomerular injury (**F**) or tubulointerstitial injury (**G**) of control, VHL^∆PT^, con/STZ and VHL^∆PT^/STZ. Arithmetic means ± SEM of n = 5-7 per group; **P* < 0.05, ****P* < 0.001. (**A**, **B**, **D**, **F** and **G**) Each point or band represents an individual mouse. Nonparametric Kruskal-Wallis with Dunn’s post test.

**
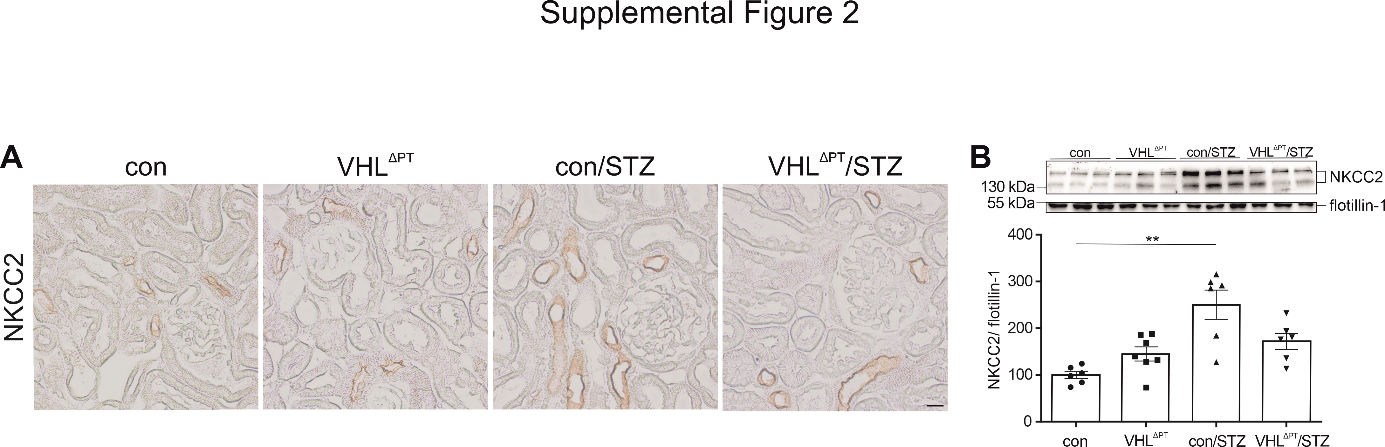
**

**Supplemental Figure 2. Localization and expression of NKCC2.** (**A**) Representative images of immunohistochemical staining of NKCC2 obtained from control, VHL^∆PT^, con/STZ and VHL^∆PT^/STZ. Scale bar = 20 µm. (**B**) Representative blot and densitometrical evaluation of western blot of NKCC2 obtained from control, VHL^∆PT^, con/STZ and VHL^∆PT^/STZ with flotillin-1 as reference. The higher expression level seemed to be independent of vasopressin-mediated phosphorylation, since p-NKCC2/NKCC2 ratio remained unaltered (data are not shown). Arithmetic means ± SEM of n = 5-7 per group; ***P* < 0.01. Each point or band represents an individual mouse. Nonparametric Kruskal-Wallis with Dunn’s post test.

**
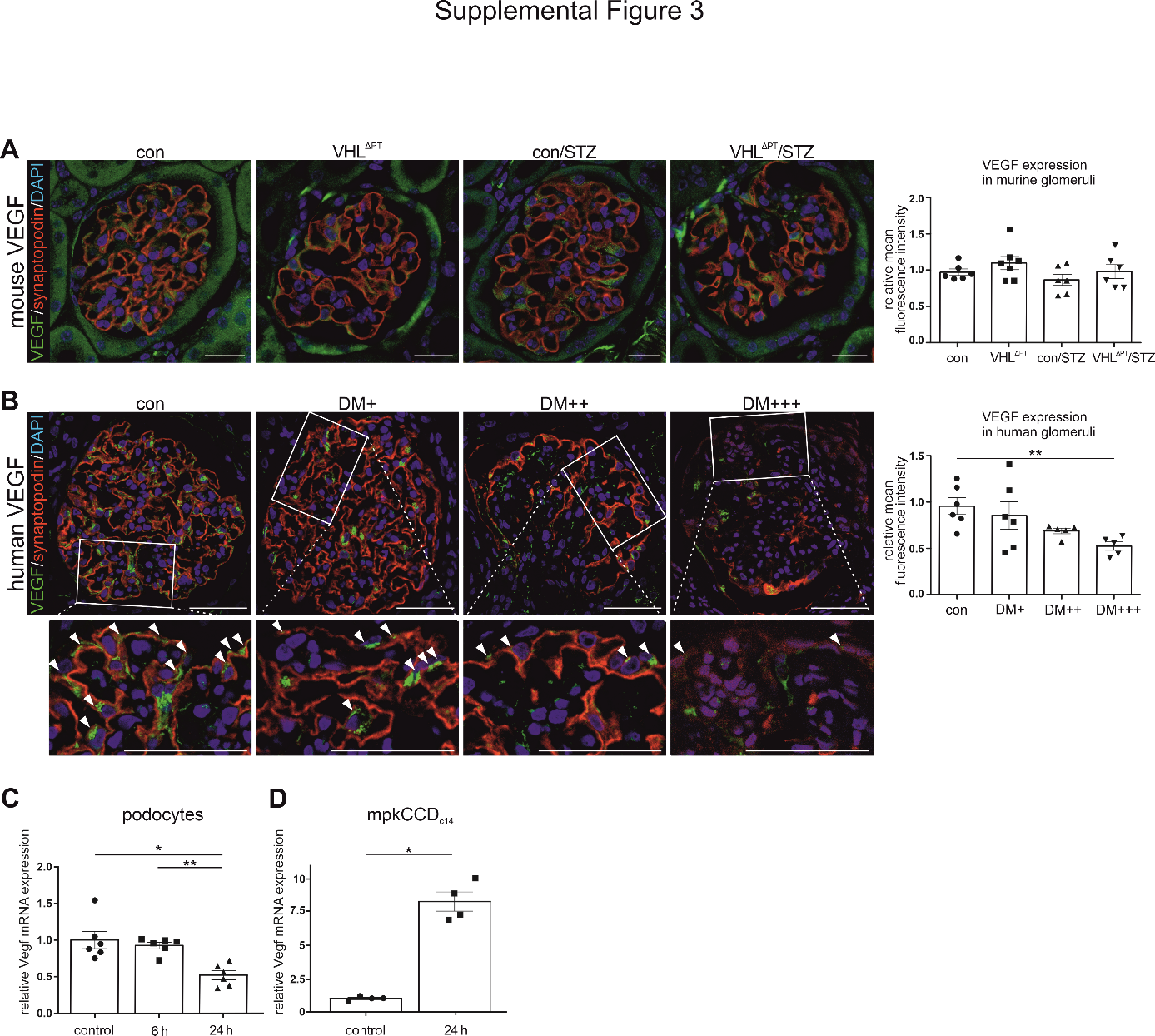
**

**Supplemental Figure 3. Glomerular VEGF expression in the mouse model and human biopsies with DKD. (A)** Representative images of immunohistochemical double staining of glomerular VEGF and synaptopodin (to visualize podocytes) in control, VHL^∆PT^, con/STZ and VHL^∆PT^/STZ. Magnification scale bar = 20 µm. Mean vascular endothelial growth factor (VEGF) fluorescence intensities obtained from control, VHL^ΔPT^, con/STZ and VHL^ΔPT^/STZ. Arithmetic means ± SEM of n = 5-7 per group. (**B**) Representative images of immunohistochemical double staining of glomerular VEGF and synaptopodin (to visualize podocytes) from human biopsies of patients with interstitial nephritis (control), mild DM (DM+), moderate DM (DM++) and severe DM (DM+++). Magnification scale bar = 50 µm. Mean VEGF fluorescence intensities obtained from mild DM (DM+), moderate DM (DM++) and severe DM (DM+++) compared to control (interstitial nephritis). Arithmetic means ± SEM of n = 5-6 per group. ***P* < 0.01. Below, detailed higher magnification image as marked in the image above. Scale bar = 50 µm. (**C**) VEGF mRNA expression of podocytes treated with high glucose and hypoxia for 6 and 24 hours. Arithmetic means ± SEM of n = 6 per group; **P* < 0.05, ***P* < 0.01. (**D**) VEGF mRNA expression of mpkCCD_c14_ cells treated with high glucose and hypoxia for 24 hours. Arithmetic means ± SEM of n = 4 per group; **P* < 0.05. Each point represents an individual mouse or patient. Nonparametric Kruskal-Wallis with Dunn’s post test.

**
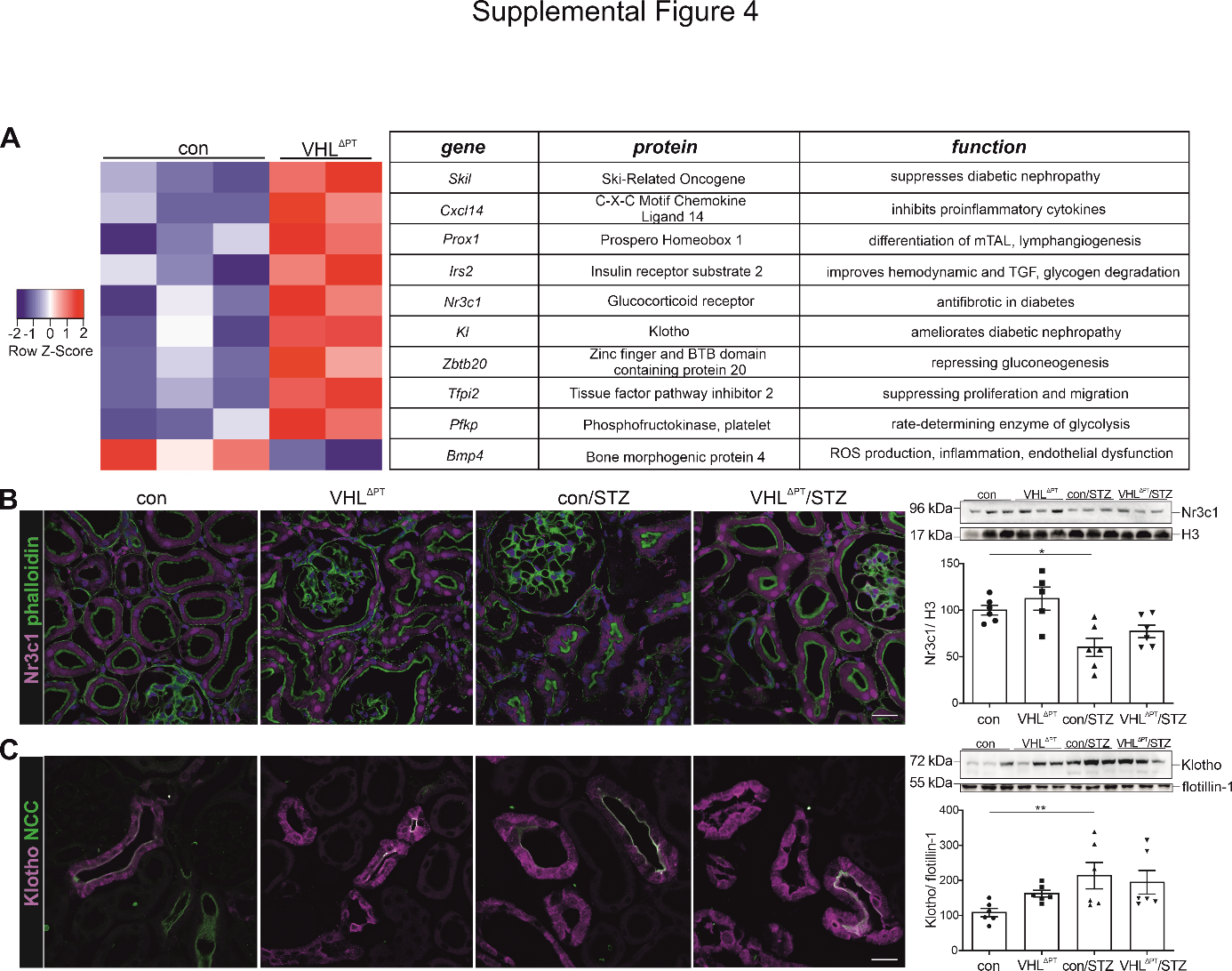
**

**Supplemental Figure 4**. **Proximal tubular *Vhl* deletion augments or reduces gene expression known to result in an amelioration of DKD.** (**A**) Heat map and table of genes significantly increased or reduced between control and VHL^∆PT^ with functions in improving diabetes outcome. Filter criteria DESeq P-values < 0.05, fold change > 1.5. (**B**) In VHL^ΔPT^, a strong glucocorticoid receptor expression was found in nuclei of PT and, to a lesser extent, in the distal tubule in comparison to controls. Scale bar = 20 µm. Arithmetic means ± SEM of n = 5-6 per group; **P* < 0.05. Western blot analysis of verified nuclear fractions revealed a slightly higher expression level in VHL^ΔPT^, and reduced expression level in con/STZ and VHL^ΔPT^/STZ Histone H3 was used as reference. (**C**) Klotho, which is expressed in the distal and connecting tubule, was increased in VHL^ΔPT^, con/STZ and VHL^ΔPT^/STZ mice with flotillin-1 as reference. Scale bar = 20 µm. Arithmetic means ± SEM of n = 5-6 per group; ***P* < 0.01. (**B** and **C**) Each point or band represents an individual mouse. Nonparametric Kruskal-Wallis with Dunn’s post test.

**
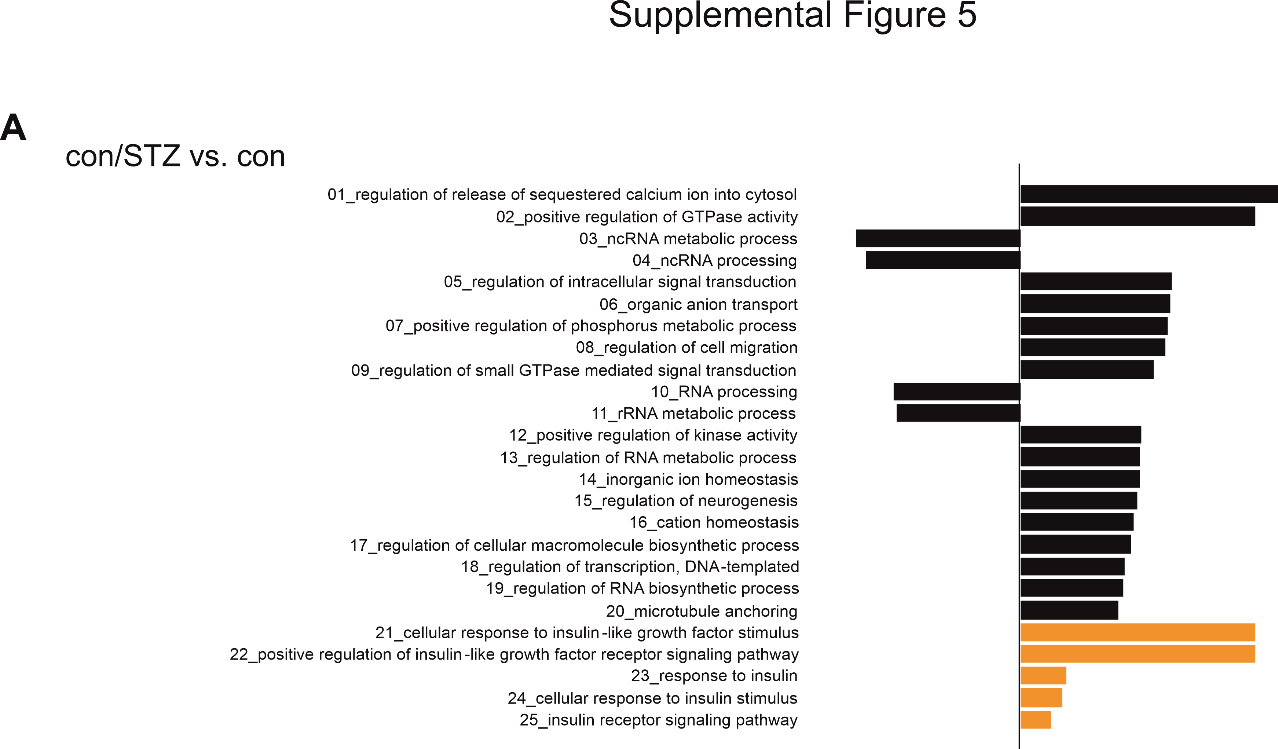
**

**Supplemental Figure 5. Gene ontology enrichment analysis of con /STZ vs. con.** Top 25 biological processes enriched/depleted in the corresponding comparisons. In addition, processes containing the term „insulin“ are shown in orange and listed.

**
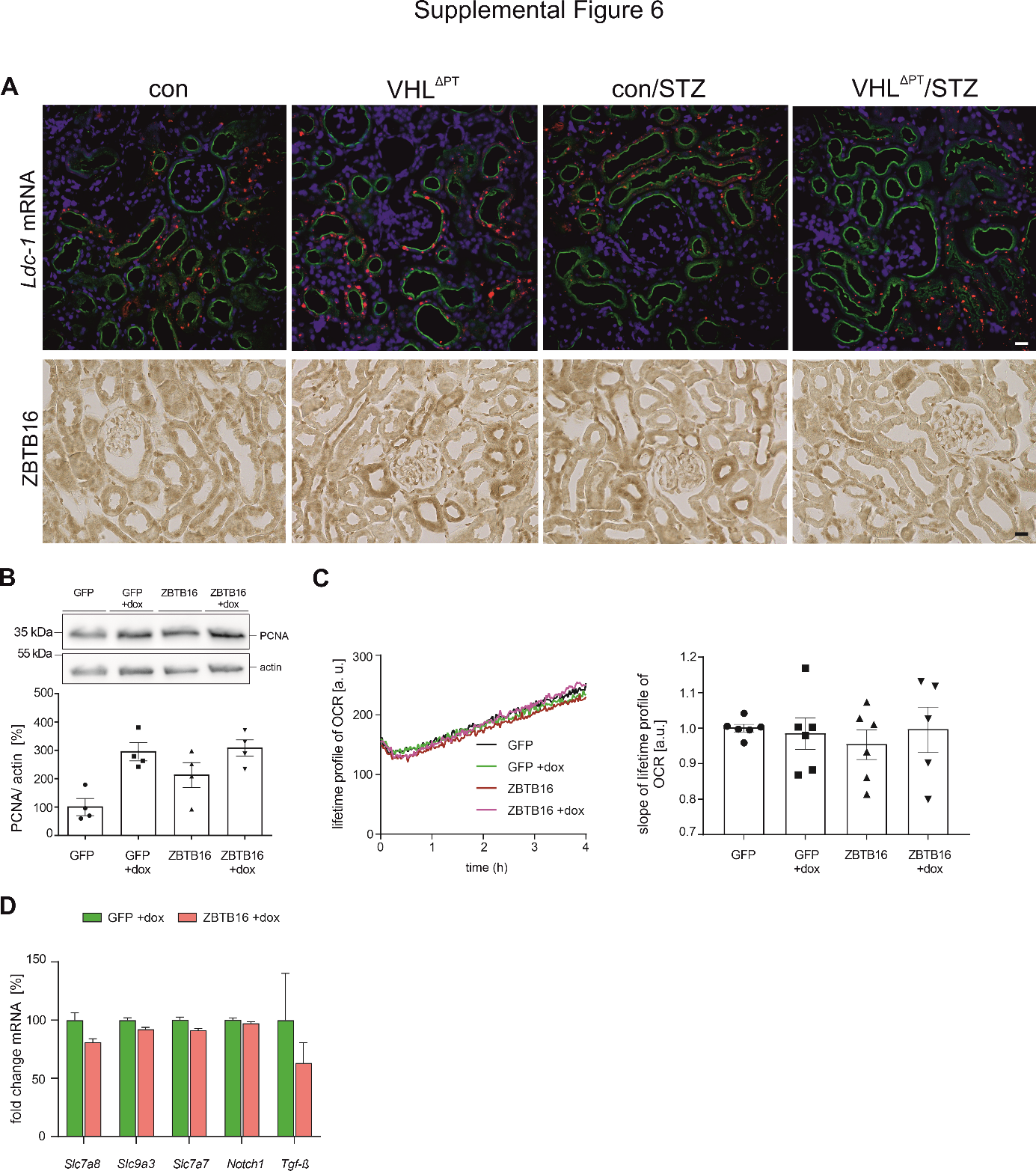
Supplemental Figure 6. Localisation of *Ldc-1* and ZBTB16 and analysis of ZBTB16 function in cell proliferation, cellular oxygen consumption and changes in transcript abundances.** (**A**) In situ hybridization for *Ldc-1* mRNA and immunohistochemistry of ZBTB16 in control, VHL^∆PT^, con/STZ and VHL^∆PT^/STZ. Magnification scale bar = 20 µm. (**B**) Western blot of nuclear fractions and densitometrical evaluation of PCNA obtained from control (GFP) and ZBTB16 overexpressing OKC with actin as reference. n = 4 independent experiments. (**C**) OCR of control (GFP) and ZBTB16 overexpressing OKC. Arithmetic means ± SEM, n = 5-6 independent experiments. (**D**) Quantitative RT-PCR analysis of *Slc7a8*, *Slc9a3*, *Slc7a7*, *Notch1* and *Tgf-ß* in control (GFP) and ZBTB16 overexpressing OKC. Arithmetic means ± SEM of n = 9 independent experiments.

**Supplemental Table 1. mRNA levels of control, VHL^∆PT^, con/STZ and VHL^∆PT^/STZ.**

mRNA sequencing performed on mRNA isolated from renal specimen of control, VHL^∆PT^, con/STZ and VHL^∆PT^/ STZ. All relevant data have been deposited in the Gene Expression Omnibus under accession number GSE210401. (please see Excel-sheet „Supplemental_Table_1.xlsx" and „SupplementalFile_for_Supplemental_Table_1.txt")

**Supplemental Table 2.** Quantitative RT-PCR primers and settings.

| Primer | Sequence (5′-3′) | Product size (bp) | PCR cycles | Annealing temperature (°C) |
| --- | --- | --- | --- | --- |
| *Gapdh for* | gaatccaccggcgtctttac | 271 | 40 | 63 |
| *Gapdh rev* | gtcttctgggtagcggtgat |  |  |  |
| *Slc7a7 for* | gccatccctgctcttcaatg | 200 |  | 62 |
| *Slc7a7 rev* | aggcagaagacaatggggaa |  |  |  |
| *Slc7a8 for* | tgggccttgctctcattgta | 189 |  | 59 |
| *Slc7a8 rev* | tggttggtggggtagatcac |  |  |  |
| *Slc9a3 for* | tctgggactcttgctcttg | 241 |  | 62 |
| *Slc9a3 rev* | aggacaatgagcagagcact |  |  |  |
| *Notch1 for* | ctcctctccatgcagctgta | 170 |  | 59 |
| *Notch1 rev* | tgcatgggagttgatcagg |  |  |  |
| *Tgf-β for* | ctaccatgccaacttctgcc | 168 |  | 63 |
| *Tgf-β rev* | cggcccacatagtagacgat |  |  |  |
