# Supplementary figures and images for: Targeted deletion of von-Hippel-Lindau in the proximal tubule conditions the kidney against early diabetic kidney disease
Source: Cell Death Dis. 2023 Aug 26;14(8):562. doi: 10.1038/s41419-023-06074-7 (PMC10457389; doi:10.1038/s41419-023-06074-7)

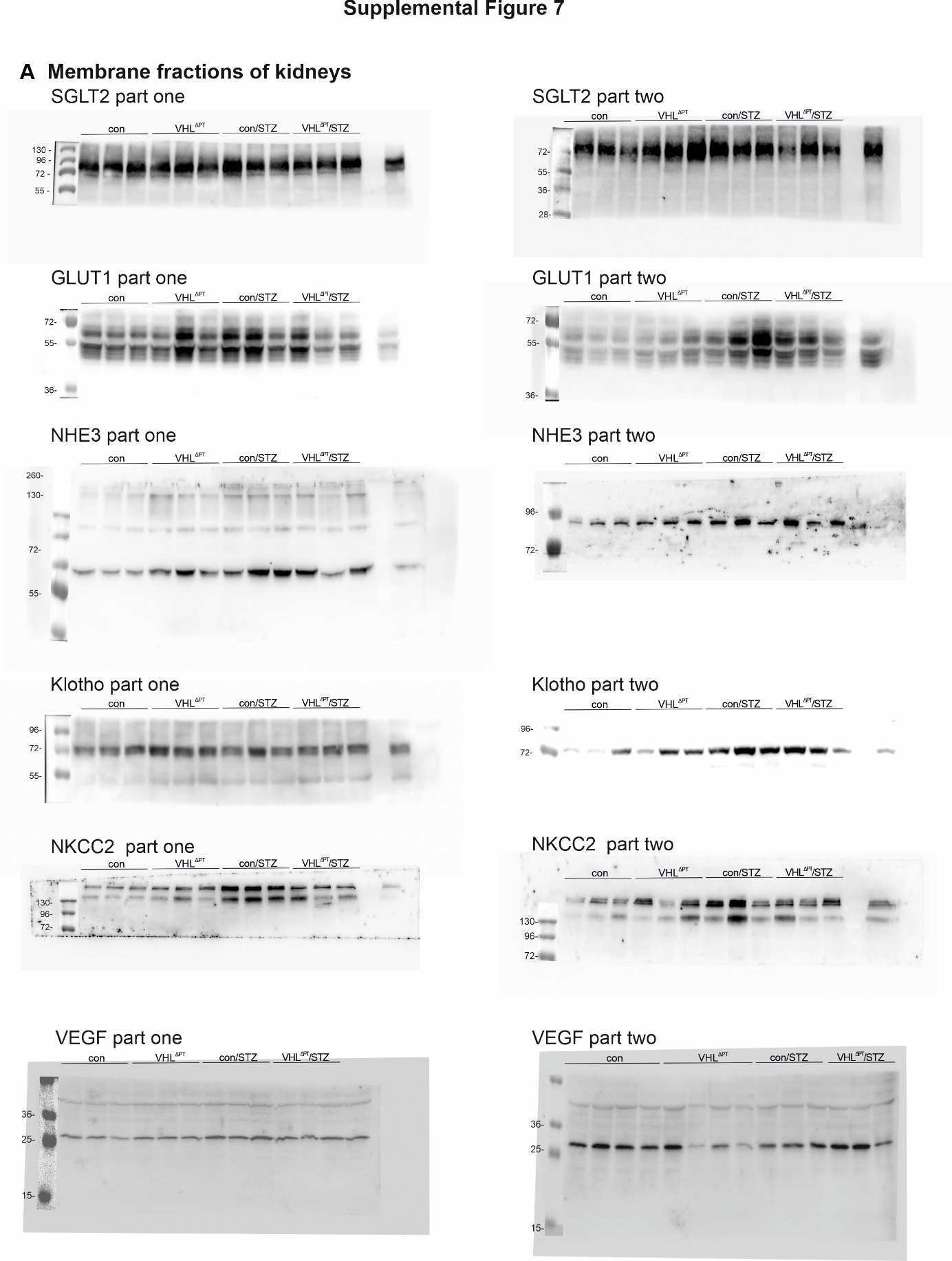

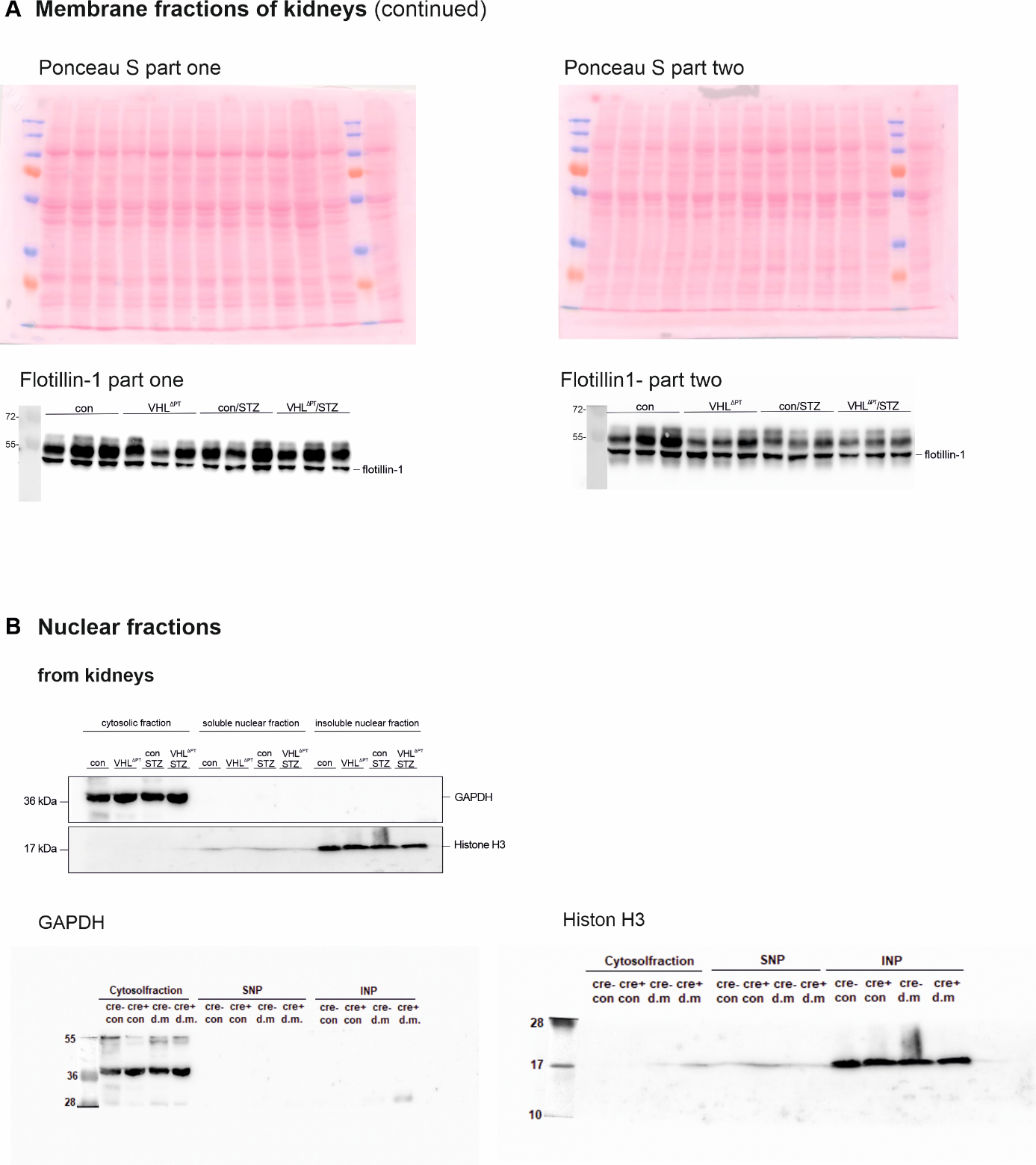

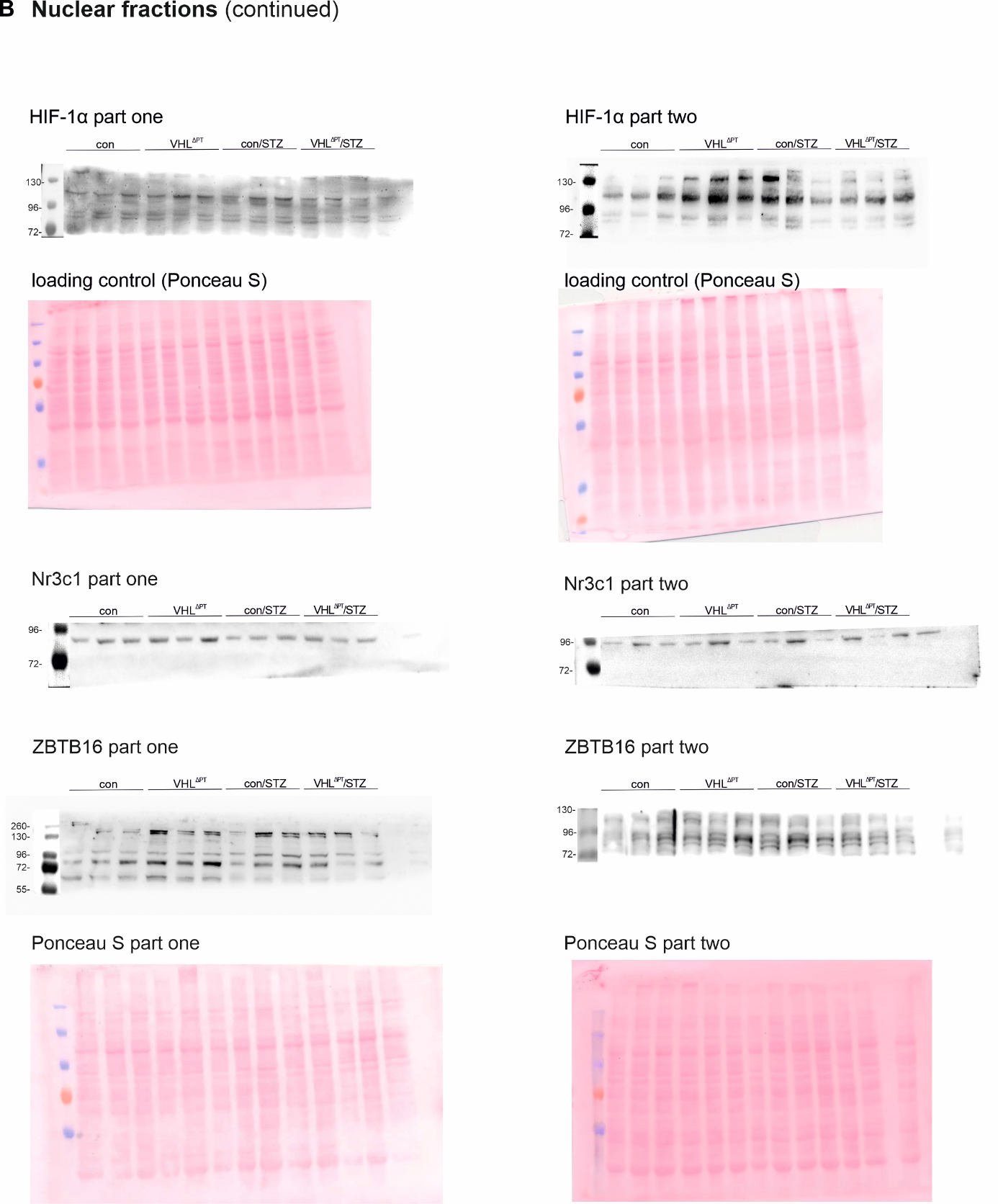

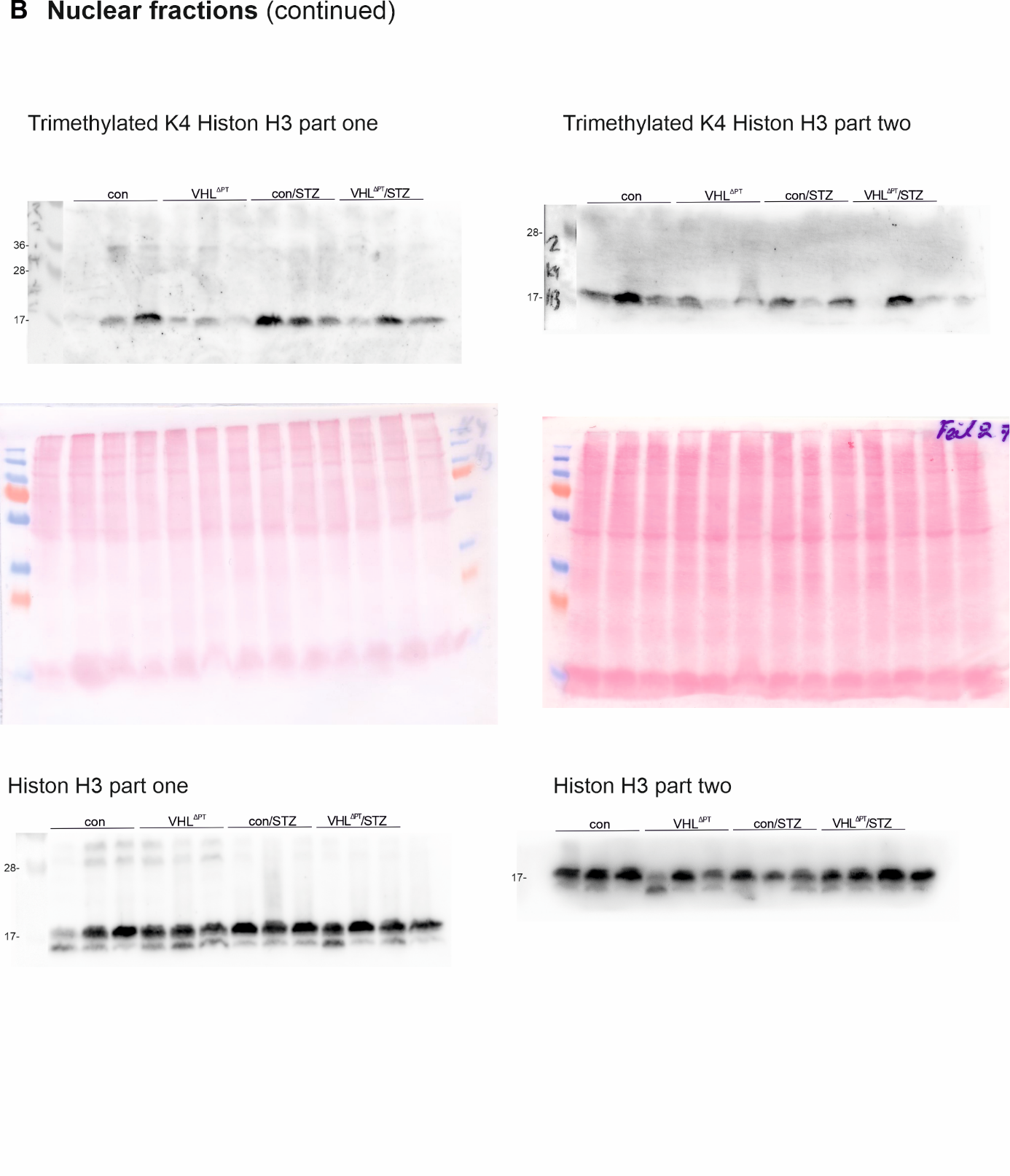

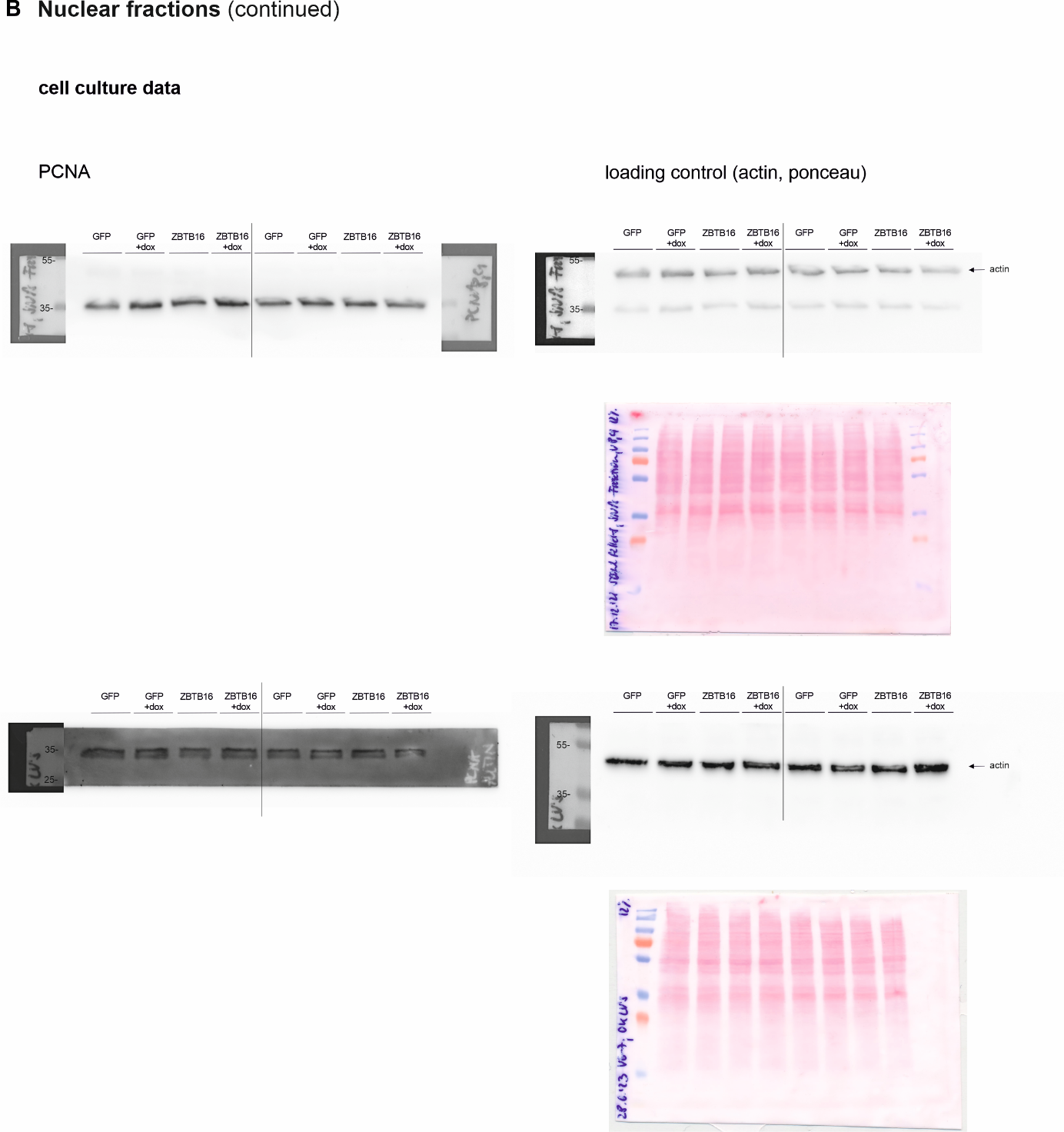

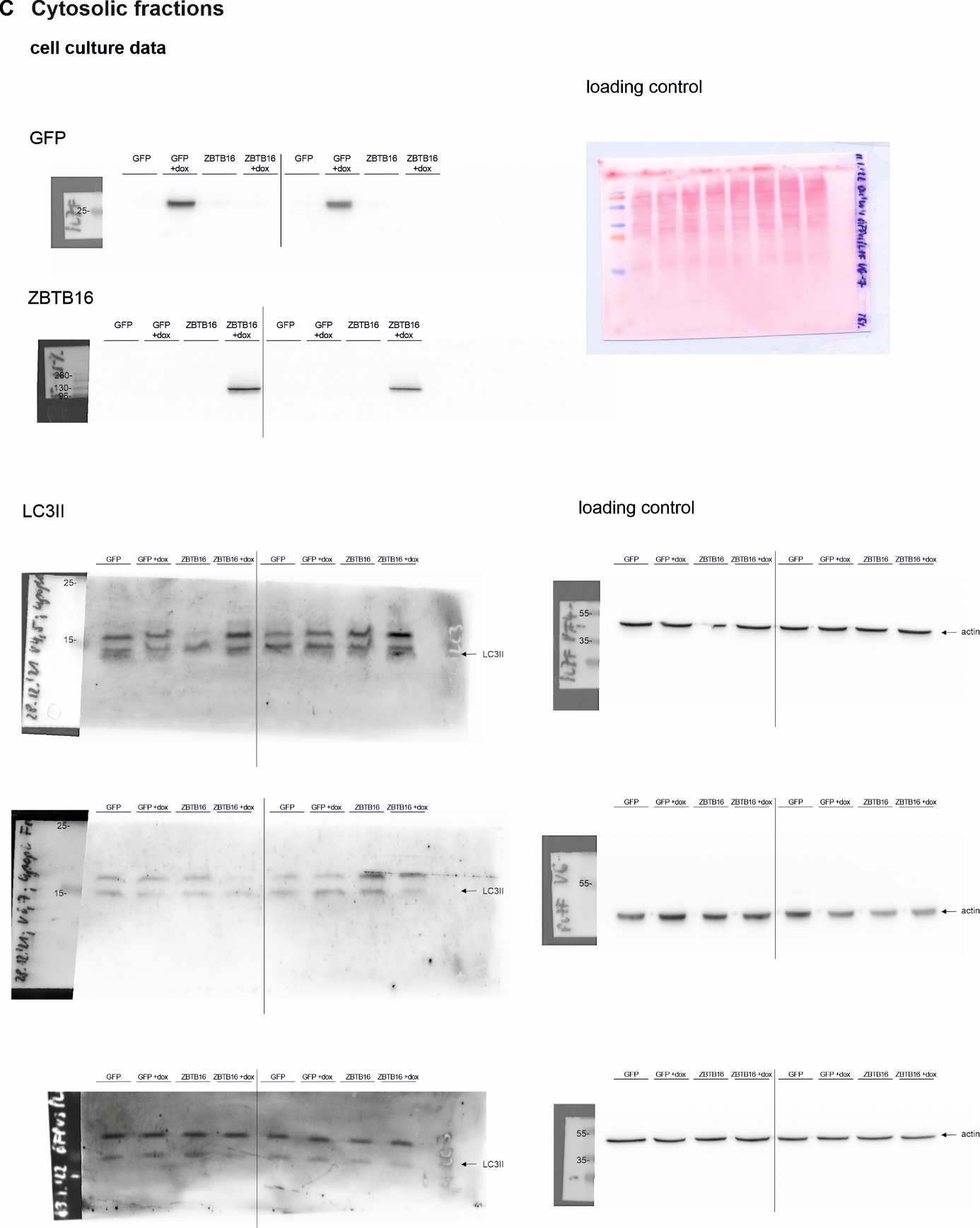

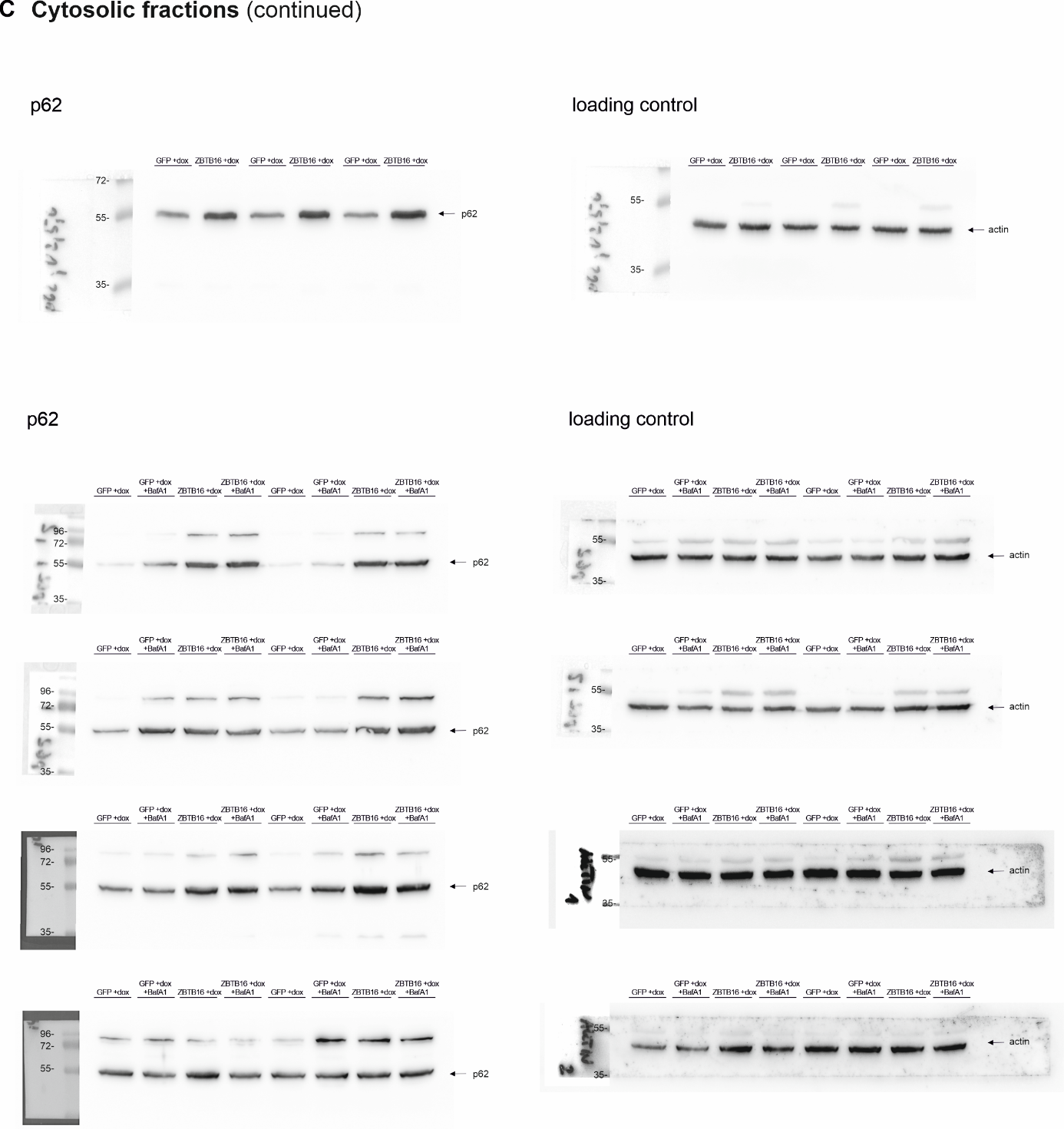

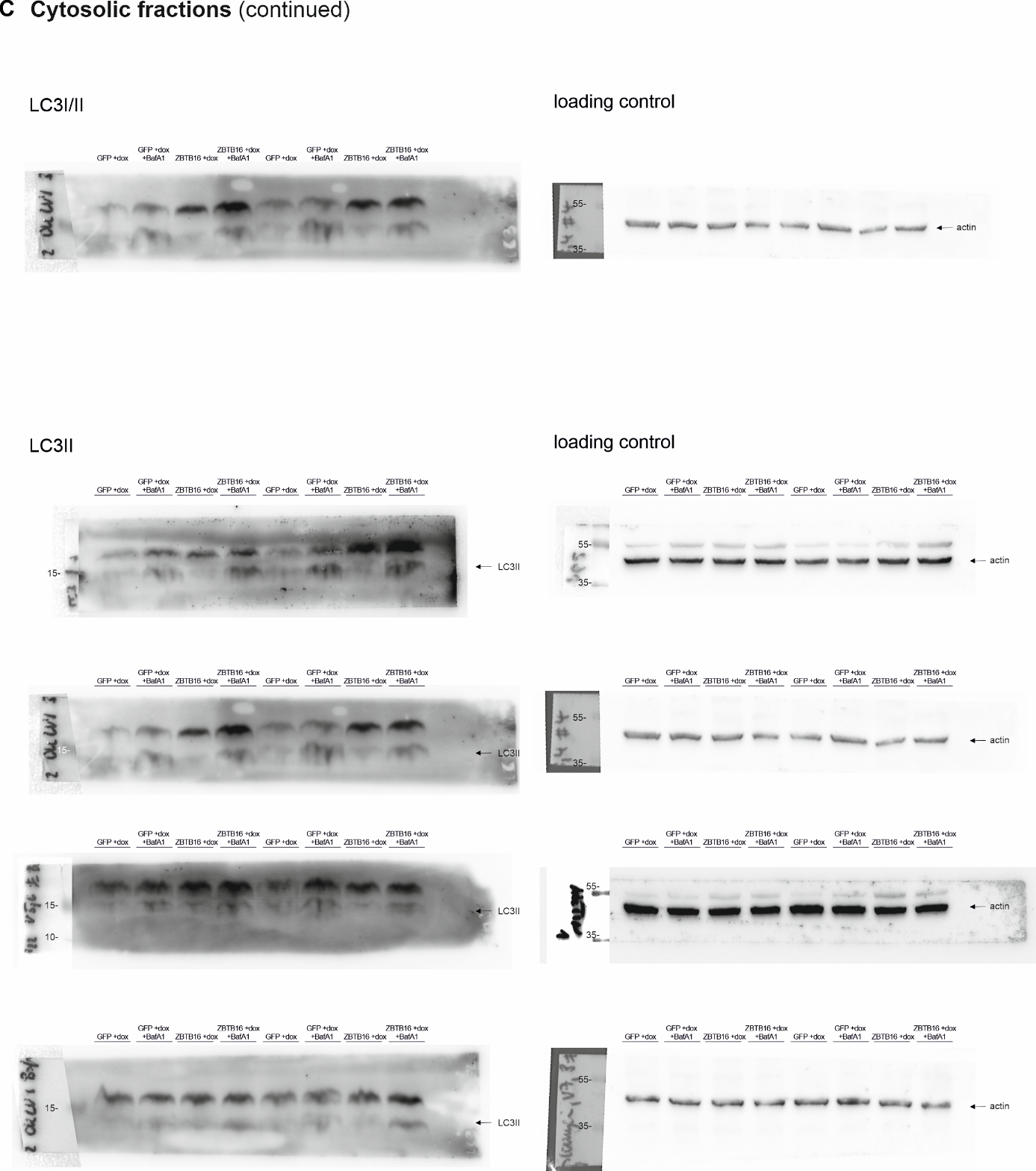

Supplement: Supplementary file 3 — uncropped western blot data [file 41419_2023_6074_MOESM3_ESM.docx]
